# NeuroD1-GPX4 signaling leads to ferroptosis resistance in hepatocellular carcinoma

**DOI:** 10.1371/journal.pgen.1011098

**Published:** 2023-12-22

**Authors:** Ping Huang, Wei Duan, Cao Ruan, Lingxian Wang, Rendy Hosea, Zheng Wu, Jianting Zeng, Shourong Wu, Vivi Kasim

**Affiliations:** 1 The Key Laboratory of Biorheological Science and Technology, Ministry of Education, College of Bioengineering, Chongqing University, Chongqing, China; 2 The 111 Project Laboratory of Biomechanics and Tissue Repair, College of Bioengineering, Chongqing University, Chongqing, China; 3 Department of Hepatobiliary and Pancreatic Oncology, Chongqing University Cancer Hospital, Chongqing University, Chongqing, China; 4 Chongqing Key Laboratory of Translational Research for Cancer Metastasis and Individualized Treatment, Chongqing University Cancer Hospital, Chongqing University, Chongqing, China; Brigham and Women’s Hospital, UNITED STATES

## Abstract

Cell death resistance is a hallmark of tumor cells that drives tumorigenesis and drug resistance. Targeting cell death resistance-related genes to sensitize tumor cells and decrease their cell death threshold has attracted attention as a potential antitumor therapeutic strategy. However, the underlying mechanism is not fully understood. Recent studies have reported that NeuroD1, first discovered as a neurodifferentiation factor, is upregulated in various tumor cells and plays a crucial role in tumorigenesis. However, its involvement in tumor cell death resistance remains unknown. Here, we found that NeuroD1 was highly expressed in hepatocellular carcinoma (HCC) cells and was associated with tumor cell death resistance. We revealed that NeuroD1 enhanced HCC cell resistance to ferroptosis, a type of cell death caused by aberrant redox homeostasis that induces lipid peroxide accumulation, leading to increased HCC cell viability. NeuroD1 binds to the promoter of *glutathione peroxidase 4* (*GPX4*), a key reductant that suppresses ferroptosis by reducing lipid peroxide, and activates its transcriptional activity, resulting in decreased lipid peroxide and ferroptosis. Subsequently, we showed that NeuroD1/GPX4-mediated ferroptosis resistance was crucial for HCC cell tumorigenic potential. These findings not only identify NeuroD1 as a regulator of tumor cell ferroptosis resistance but also reveal a novel molecular mechanism underlying the oncogenic function of NeuroD1. Furthermore, our findings suggest the potential of targeting NeuroD1 in antitumor therapy.

## Author summary

Aberrant expression of cell death resistance-related genes is common in tumor cells and contributes to their resistance to cell death and tumorigenesis. Transcription factor NeuroD1 has been found to be highly expressed in various tumors and could promote tumorigenesis. However, whether it is involved in tumor cell death resistance remains unclear. In this study, we demonstrate that NeuroD1 enhances the resistance of hepatocellular carcinoma (HCC) cells to ferroptosis. Knocking down *NeuroD1* leads to the increase of reactive oxygen species (ROS), toxic lipid peroxides, as well as DNA and mitochondrial damages. These subsequently trigger ferroptosis. We further revealed that NeuroD1 acts as a transcriptional activator of glutathione peroxidase 4 (*GPX4*), a key molecule that reduces lipid peroxide and thus protects cells against ferroptosis, thereby upregulates the expression level of GPX4 and suppresses HCC cells ferroptosis. Our results also confirmed the critical role of NeuroD1/GPX4 in tumorigenesis *in vivo*. Together, our findings reveal *NeuroD1* as a crucial ferroptosis suppressor gene, thereby unraveling a novel mechanism of tumor cells ferroptosis resistance. Furthermore, our results suggest that targeting NeuroD1 might be a potential antitumor therapeutic strategy.

## Introduction

Cell death resistance is one of the hallmarks of cancer [1]. The balance of cell death plays a vital role in regulating cell population size and tumorigenesis. Cell death can be classified as accidental cell death (ACD), a biologically uncontrolled process, and regulated cell death (RCD), which is characterized by controlled signaling pathways [2]. RCD includes apoptosis, necroptosis, autophagy, and ferroptosis, which can occur in the presence or absence of exogenous environmental or intracellular perturbations [3]. Tumor cells can escape the RCD route by evolving various mechanisms that lead to increased cell death thresholds [4]. Aberrant expression of RCD-related genes, such as the caspase and Bcl-2 families, due to mutations or impaired regulatory mechanisms is frequently observed in tumor cells, leading to an elevation in the cell death threshold [5–8]. Cell death resistance is involved in every step of tumorigenesis. At the tumor initiation stage, mutations in tumor suppressor genes such as *TP53* and *BRCA1/2* block cell death induced by DNA damage, leading to the initiation of tumor formation [9]. With an increase in tumor mass, the tumor microenvironment becomes more depleted of nutrients and oxygen; however, cell death resistance helps tumor cells endure such a severe microenvironment [10]. In the metastatic stage, cell death resistance enables tumor cells to survive and proliferate under unfamiliar conditions by circumventing the limitations of growth factor signaling [11]. Hence, cell death resistance plays a key role in tumor initiation and development. Furthermore, cell death resistance impedes an effective response to cancer therapy as it leads to high tumor survival potential and therapeutic resistance [12]. Therefore, combining therapeutic strategies targeting tumor cell death resistance with conventional antitumor therapies has attracted attention as a potential therapeutic strategy [3, 13].

The RCD pathway is believed to function as a natural barrier against malignancy; however, the emergence of chemotherapy resistance during cancer therapy has been a major problem due to cell death resistance [14, 15]. Recent studies have reported that not only apoptosis but also other types of cell death, including necroptosis, ferroptosis, and autophagic cell death, are closely related to tumor progression, poor prognosis, and drug resistance. Apoptosis is a non-inflammatory cell death process that eliminates damaged cell [16, 17]. Aberrant overactivation of apoptosis resistance-related genes, such as Bcl-2 family and genes in PTEN/P13K/AKT pathway, are frequently found in cancer cells [18, 19]. Necroptosis exhibits morphological features similar to necrosis and can be triggered by various cytokines or pattern recognition receptors (PRRs), such as tumor necrosis factor-α (TNF-α), which initiate necroptosis by promoting receptor interacting serine/threonine kinase 3 (RIPK3)-mediated mixed lineage kinase domain-like pseudokinase (MLKL) phosphorylation [20–22]. However, some primary tumors and cancer cell lines, such as melanoma, breast cancer, and colorectal cancer, exhibit minimal or no RIPK3 activity, leading to enhanced resistance to necroptosis [23, 24]. Autophagic cell death, induced by nutrient deficiency and abnormal protein accumulation, is characterized by increased cell-substrate adhesion, broken or disappeared endoplasmic reticulum structures, and focal swelling of the perinuclear space [25, 26]. In tumor cells, autophagic cell death is suppressed by the highly expressed phosphoglycerate kinase (PGK1), which suppresses the expression of microtubule-associated protein light chain 3II (LC3-II) and the formation of autophagosomes through binding and phosphorylating proline-rich Akt substrate 40 kDa (PRAS40) [27, 28]. Ferroptosis is a form of RCD that depends on iron (Fe^2+^)-mediated lipid peroxidation caused by the disruption of redox homeostasis [29]. Decreased ferroptosis rates and increased ferroptosis resistance have been observed in various cancer types [30]. Upregulation of genes involved in ferroptosis resistance, such as solute carrier family 7 member 11 (*SLC7A11*) and glutathione peroxidase 4 (*GPX4)*, as well as downregulation of polyunsaturated fatty acid (PUFA)-containing PLs (PUFA-PLs), which are key catalytic enzyme substrates in ferroptosis, have been linked to ferroptosis evasion and enhanced tumor growth [30, 31]. Therefore, the resistance of tumor cells to cell death presents a significant barrier and is one of the major challenges in achieving curative antitumor therapy.

Neurogenic differentiation factor 1 (NeuroD1) is a member of the basic helix-loop-helix transcription factor family that plays a critical role in inducing neuronal developmental programs by promoting neurogenic differentiation [32, 33]. NeuroD1 could also reprogram other cell types into neurons. For example, it can boost the transcriptional activity of scratch family transcriptional repressor 1 (Scrt1) and Meis homeobox 2 (Meis2) to convert microglia into neurons [34], and can directly reprogram glial cells into neurons while boosting their circuitry integration [35]. Furthermore, it promotes neuronal migration by inducing the genes involved in epithelial-to-mesenchymal transformation [36, 37]. Recent studies have shown that NeuroD1 is highly expressed in various tumor cells and is closely associated with tumorigenesis and poor prognosis. High expression of NeuroD1 can promote neuroblastoma formation [38]. Furthermore, it can enhance the migration potential of small cell lung cancer cells and pancreatic cancer cells [39, 40]. Our recent studies also showed that NeuroD1 promotes tumorigenesis by downregulating the p53/p21 axis, thereby promoting cell cycle progression, and by upregulating glucose-6-phosphate dehydrogenase (G6PD) expression, thereby enhancing glucose metabolic reprogramming in tumor cells [41, 42]. However, the role of NeuroD1 in tumorigenesis and its potential involvement in tumor cell death resistance are not yet fully understood.

In this study, to explore the role of NeuroD1 in tumor cell death resistance, we first examined the type of cell death triggered by knocking down *NeuroD1* using various cell death inhibitors, and found that ferroptosis inhibitors significantly abolished the increase in cell death induced by *NeuroD1* knockdown. Using *in silico*, *in vitro*, and *in vivo* analyses, we further revealed the role of NeuroD1 in suppressing tumor cells ferroptosis, and elucidated the molecular mechanism underlying this regulation. We revealed that NeuroD1 promotes the transcriptional activity of the *GPX4* promoter, leading to an increase in ferroptosis resistance, and subsequently, tumorigenesis. Our results not only link NeuroD1 with tumor cell ferroptosis resistance but also reveal an unprecedented regulatory pathway of ferroptosis. Furthermore, these findings suggest a potential antitumor therapeutic strategy targeting NeuroD1.

## Results

### *NeuroD1* knockdown induces cell death

**Fig 1:**
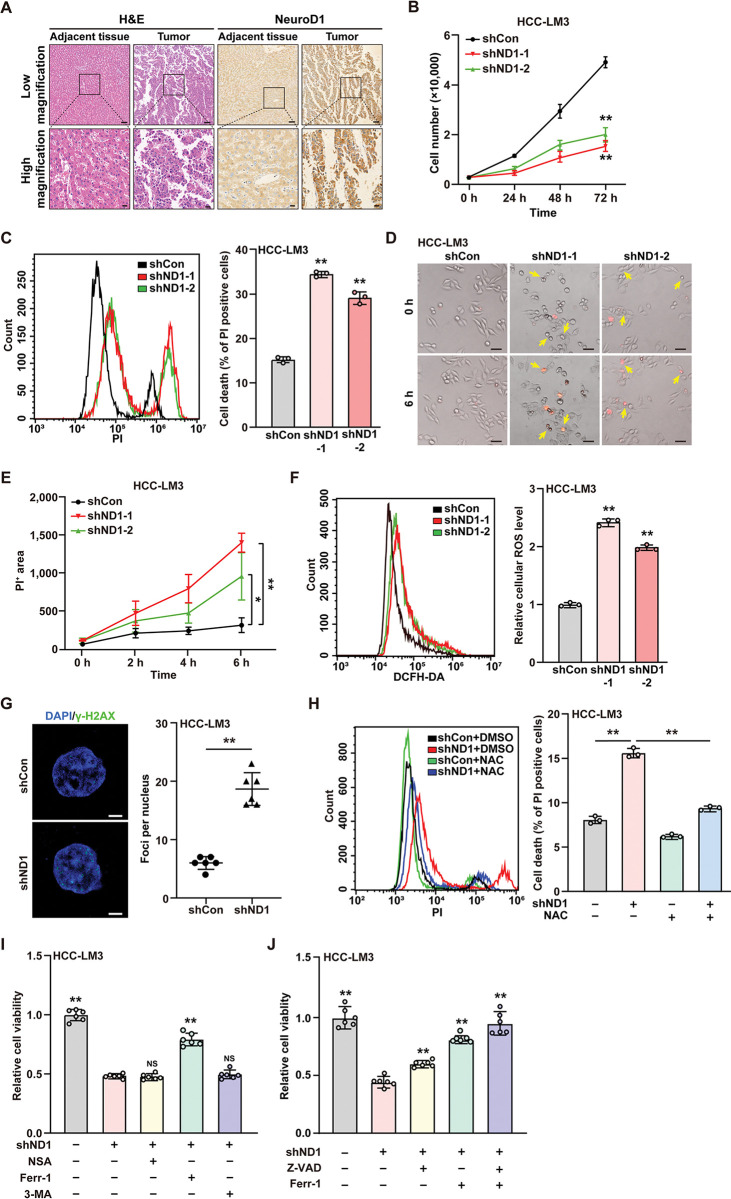
NeuroD1 increases HCC cell death resistance. **(A)** Immunohistochemistry staining showing the expression level of NeuroD1 in clinical human HCC and corresponding adjacent tissues. (top panels: low magnification, scale bars:100 μm; bottom panels: high magnification, scale bars: 20 μm) (**B)** Viability of *NeuroD1*-knocked down HCC-LM3 cells (n = 6). (**C)** Cell death rate of *NeuroD1*-knocked down HCC-LM3 cells, as examined using PI staining and flow cytometry. (**D–E)** Time-lapse images of PI-positive *NeuroD1*-knocked down HCC-LM3 cells. Representative images (D; scale bars: 40 μm) and quantification results of PI fluorescence intensity (E; *F* value = 21.99) are shown. Yellow arrows: PI-positive cells. **(F)** Total cellular ROS level in *NeuroD1*-knocked down HCC-LM3 cells, as examined using DCFH-DA staining and flow cytometry. (**G)** Expression level of γ-H2AX in *NeuroD1*-knocked down HCC-LM3 cells, as analyzed using Immunofluorescent staining. Representative images (left; scale bars: 5 μm) and quantification results (right; n = 6) are shown. **(H)** Cell death rate of *NeuroD1*-knocked down HCC-LM3 cells treated with 5 μM N-acetylcysteine (NAC), as examined using PI staining and flow cytometry (*F* value = 371). (**I)** Relative viability of *NeuroD1*-knocked down HCC-LM3 cells treated with Ferr-1 (final concentration: 5 μM), NSA (final concentration: 5 μM), and 3-MA (final concentration: 20 μM) for 48 h (n = 6; *F* value = 226.6). (**J)** Relative viability of *NeuroD1*-knocked down HCC-LM3 cells treated with Ferr-1 (final concentration: 5 μM) and/or Z-VAD (final concentration: 20 μM) for 48 h (n = 6; *F* value = 68.89). Cells transfected with shCon and/or treated with DMSO were used as controls. Quantification data are expressed as mean ± SD (n = 3; unless otherwise indicated). *P* values were calculated using two-tailed unpaired Student’s *t*-test, or using one-way ANOVA and Tukey multiple comparisons when more than two groups were compared. shND1: shRNA expression vector targeting *NeuroD1*; *P < 0.05; **P < 0.01; NS: not significant.

To investigate the role of NeuroD1 in hepatocellular carcinoma (HCC), we first analyzed its expression levels in clinical HCC samples. As shown in Fig 1A, NeuroD1 expression was significantly higher in tumor lesions than in adjacent tissues, and was localized in both cytoplasm and nucleus. Next, we confirmed the efficacy of the two shRNA expression vectors targeting *NeuroD1* in HCC-LM3 cells by assessing NeuroD1 mRNA expression levels (S1A Fig). These results were further confirmed by the NeuroD1 protein expression level in HCC-LM3 and MHCC-97H cells (S1B Fig). The results showed that shND1-1 had a stronger suppressive effect; thus, we used this vector in further experiments. Furthermore, we also confirmed the efficacy of *NeuroD1* overexpression vector in HCC-LM3 and MHCC-97H cells (S1C Fig). We examined the effects of altering NeuroD1 expression on the viability and colony potential of these cell lines. Knocking down *NeuroD1* significantly suppressed the viability (Figs 1B and S2A) and colony formation potential (S2B and S2C Fig) of HCC-LM3 and MHCC-97H cells, while *NeuroD1* overexpression robustly increased them (S2D–S2G Fig). These results suggest that NeuroD1 enhances HCC cell viability.

Cell death resistance is a crucial hallmark of tumorigenesis and enhances tumor cell viability. The flow cytometry results showed that the percentage of PI-positive cells robustly increased in *NeuroD1*-knocked down HCC cells (Figs 1C and S3A) and decreased in *NeuroD1*-overexpressed cells (S3B and S3C Fig). Furthermore, as observed using time-lapse microscopy, the number of PI-positive *NeuroD1*-knocked down HCC-LM3 cells increased in a time dependent manner (Fig 1D and 1E). Together, these results indicated that NeuroD1 may be involved in HCC cell death resistance. We also found that knocking down *NeuroD1* significantly increased cellular ROS levels in HCC-LM3 cells (Fig 1F), while overexpressing *NeuroD1* robustly suppressed this effect (S3D Fig). Meanwhile, overexpressing *NeuroD1* in *NeuroD1*-knockdown HCC-LM3 cells canceled the increase of cell death rate and cellular ROS levels (S3E and S3F Fig). Furthermore, we observed increased DNA damage level in *NeuroD1*-knocked down HCC-LM3 cells (Fig 1G). Moreover, addition of the ROS inhibitor N-acetylcysteine (NAC) inhibited the increase in PI-positive cells as well as cellular ROS level in *NeuroD1*-knocked down HCC-LM3 cells (Figs 1H and S3G). Taken together, these results show that NeuroD1 can increase tumor cell resistance against cell death.

The increase of cellular ROS level, which could induce DNA damage, has been known to trigger apoptosis [14]. Hence, we next treated *NeuroD1*-knocked down cells with a pan-caspase inhibitor Z-VAD-fmk (Z-VAD) to inhibit apoptosis. However, while Z-VAD restored the viability of *NeuroD1*-knocked down HCC-LM3 cells, the effect was slight and it failed to restore the viability of those cells to the level of control (S3H Fig), indicating that while knocking down *NeuroD1* could induce apoptosis in HCC cells, it might also have triggered non-apoptotic cell death.

As increased of ROS and DNA damage could also induce necroptosis, ferroptosis, and autophagic cell death [43–45], we next treated *NeuroD1*-knocked down HCC-LM3 cells with other three inhibitors, necroptosis inhibitor necrosulfonamide (NSA), ferroptosis inhibitor ferrostatin-1 (Ferr-1), and autophagic cell death inhibitor 3-methyladenine (3-MA). As shown in Fig 1I, treatment with ferrostatin-1 had a more potent effect on restoring cell viability suppressed by knocking down *NeuroD1*. Meanwhile, NSA and 3-MA treatments did not significantly affect the viability of *NeuroD1*-knocked down HCC-LM3 cells. These results indicate that NeuroD1 might not be involved in necroptosis and autophagic cell death resistance in HCC cells; instead, it might be involved in ferroptosis resistance. We next treated HCC-LM3 cells with either Z-VAD and Ferr-1 alone, or both of them. The results showed that treatment with Ferr-1 restored the viability of *NeuroD1*-knocked down HCC-LM3 cells more significant than treatment with Z-VAD, while treatment with both of them restored the viability of these cells to a level similar to that of control (Fig 1J). These results suggest that while NeuroD1 negatively regulate apoptosis and ferroptosis in HCC cells, it contributes to tumor cell death resistance mainly by suppressing ferroptosis.

### NeuroD1 suppresses tumor cell ferroptosis

**Fig 2:**
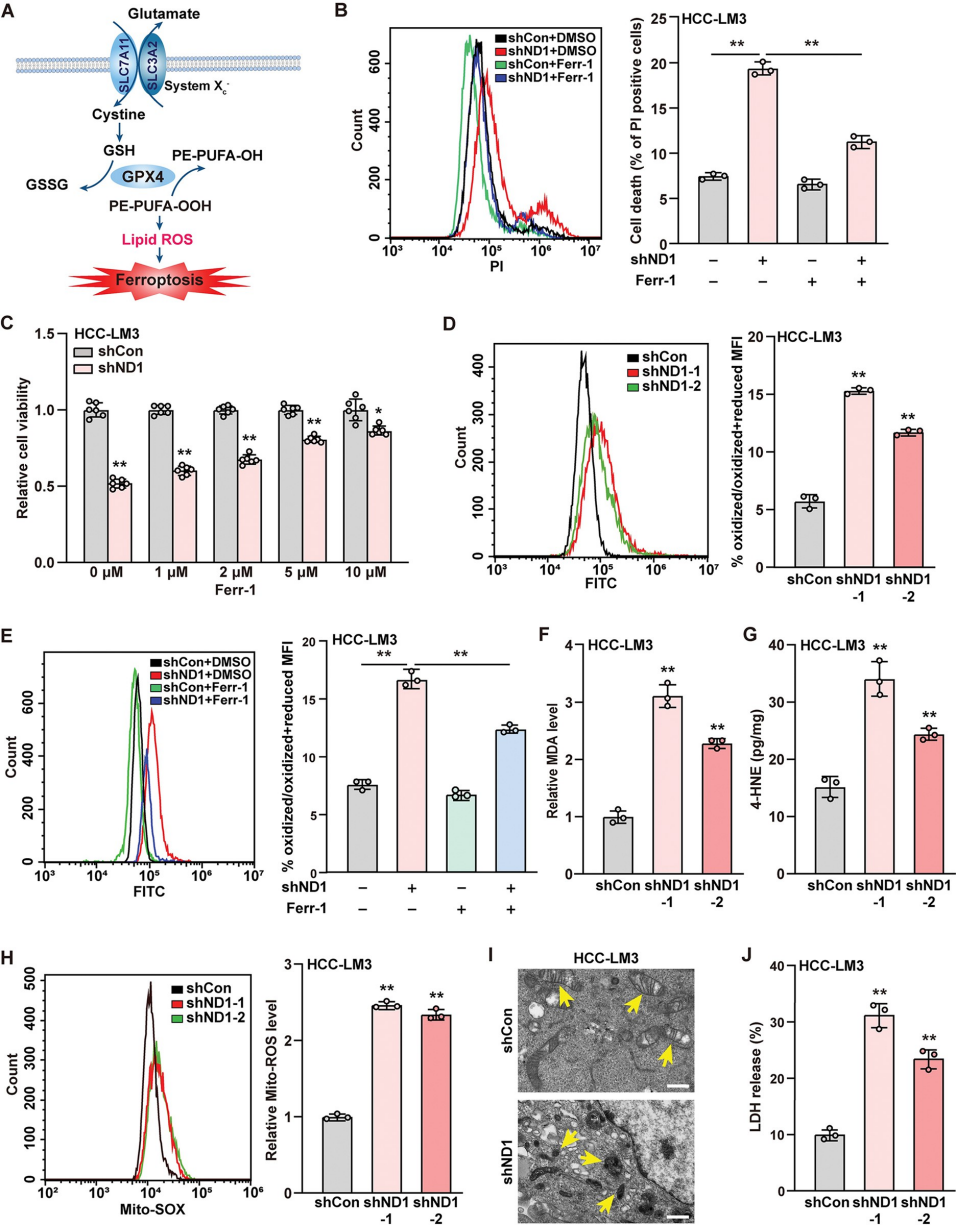
NeuroD1 inhibits HCC cell ferroptosis. **(A)** Schematic diagram of ferroptosis regulatory pathway. **(B)** Cell death rate of *NeuroD1*-knocked down HCC-LM3 cells treated with 5 μM Ferr-1 for 24 h, as examined using PI staining and flow cytometry. **(C)** Relative viability of *NeuroD1*-knocked down HCC-LM3 cells treated with indicated concentrations of Ferr-1 for 48 h (n = 6; *F* value = 331.9). **(D–E)** Lipid peroxidation level in *NeuroD1*-knocked down HCC-LM3 cells (D) and *NeuroD1*-knocked down HCC-LM3 cells treated with 5 μM Ferr-1 for 24 h (E; *F* value = 1333), as assessed using flow cytometry. Data were presented as % of oxidized/oxidized + reduced MFI. **(F–G)** MDA (F) and 4-HNE (G) levels in *NeuroD1*-knocked down HCC-LM3 cells. **(H)** Mitochondrial ROS level in *NeuroD1*-knocked down HCC-LM3 cells, as assessed by Mito-SOX staining and flow cytometry. **(I)** Mitochondrial morphology in *NeuroD1*-knocked down HCC-LM3 cells, as examined using transmission electron microscope. Yellow arrows: mitochondria. Scale bars: 1 μm. **(J)** Level of LDH released from *NeuroD1*-knocked down HCC-LM3 cells. Cells transfected with shCon and/or treated with DMSO were used as controls. Total protein was used for normalizing MDA and 4-HNE levels. Quantification data are expressed as mean ± SD (n = 3; unless otherwise indicated). *P* values were calculated using two-tailed unpaired Student’s *t*-test, or using one-way ANOVA and Tukey multiple comparisons when more than two groups were compared. shND1: shRNA expression vector targeting *NeuroD1*; *P < 0.05; **P < 0.01.

Ferroptosis is a recently discovered form of programmed cell death induced by aberrant iron ions, which leads to increased lipid peroxidation, and imbalanced cellular redox homeostasis due to the defect in glutathione (GSH) synthesis and GPX4 expression (Fig 2A). This in turn triggers mitochondrial disruption, leading to mitochondrial shrinkage and a decrease in mitochondria cristae [46]. Subsequently, lipid peroxidation triggers cellular membrane disruption, causing the release of cell contents such as lactate dehydrogenase (LDH) [47]. To confirm whether NeuroD1 regulates HCC cell viability by suppressing ferroptosis, we examined the effect of ferrostatin-1, a ferroptosis inhibitor, on cell death and viability of HCC-LM3 cells. While the knockdown of *NeuroD1* increased HCC-LM3 cell death, ferrostatin-1 treatment abolished this effect (Fig 2B). Consequently, ferrostatin-1 treatment increased the viability of *NeuroD1*-knocked down HCC-LM3 cells in a dose-dependent manner (Fig 2C). These results indicate that NeuroD1 suppresses ferroptosis in HCC cells.

Next, we investigated the effects of NeuroD1 on ferroptosis characteristics. Knocking down *NeuroD1* led to an increase in lipid ROS in HCC-LM3 and MHCC-97H cells (Figs 2D and S4A), while overexpressing *NeuroD1* robustly suppressed this effect (S4B and S4C Fig). Meanwhile, ferrostatin-1 treatment abolished the effect of *NeuroD1* knockdown on HCC-LM3 lipid ROS (Fig 2E). Furthermore, alterations in *NeuroD1* expression in HCC-LM3 cells negatively regulated the levels of malondialdehyde (MDA; Figs 2F and S4D) and 4-hydroxynonenal (4-HNE; Figs 2G and S4E), the two main products of PUFA peroxidation. Together, these data indicate the negative regulation of NeuroD1 in tumor cell ferroptosis.

Next, we examined the effect of NeuroD1 on mitochondria in HCC-LM3 cells. Knocking down *NeuroD1* promoted mitochondrial ROS levels (Fig 2H), while overexpressing *NeuroD1* suppressed them (S4F Fig). Furthermore, mitochondrial shrinkage and a reduction in mitochondrial cristae were observed in *NeuroD1*-knocked down HCC-LM3 cells (Fig 2I). Analysis of the level of LDH released into the medium revealed that *NeuroD1* alteration negatively regulated the level of LDH released from HCC-LM3 cells, suggesting that knocking down *NeuroD1* elevated cellular membrane disruption (Figs 2J and S4G). Moreover, re-expressing *NeuroD1* in *NeuroD1*-knocked down HCC-LM3 cells abolished the increase in lipid ROS, mitochondrial ROS, MDA, 4-HNE, and LDH levels caused by *NeuroD1* knockdown to the levels similar to those of control (S5A–S5E Fig). Taken together, these results indicated that NeuroD1 suppresses ferroptosis in HCC cells, thereby increasing their viability.

### NeuroD1 positively regulates GPX4 expression by enhancing its transcription

**Fig 3:**
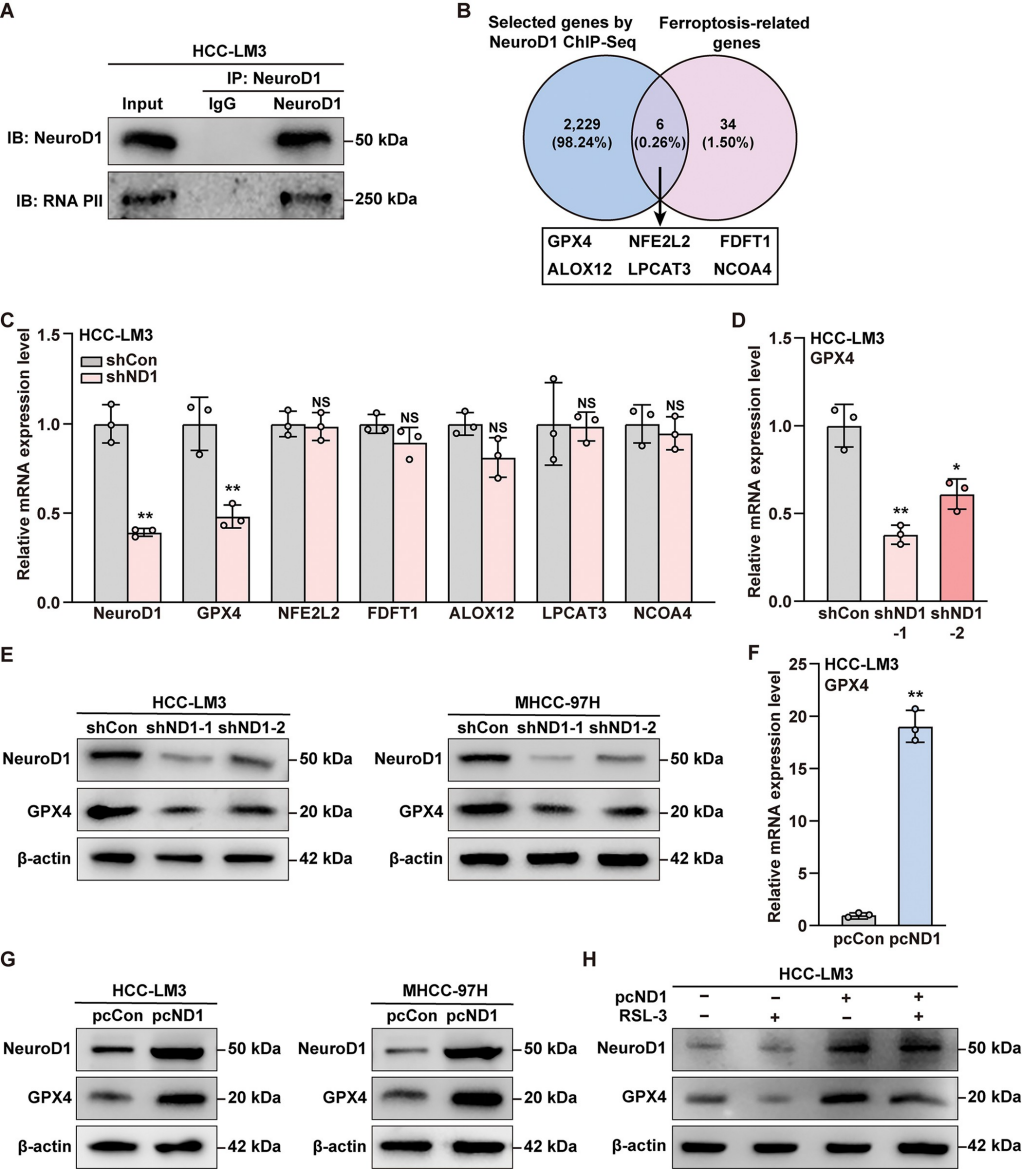
NeuroD1 positively regulates GPX4. **(A)** Physical interactions between endogenous NeuroD1 and RNA polymerase II protein in HCC-LM3 cells, as determined by anti-RNA polymerase II immunoblotting of cell lysate immunoprecipitated with anti-NeuroD1 antibody. **(B)** Overlapping of ferroptosis-related genes and NeuroD1 potential transcriptional target as identified by ChIP-seq previously reported. **(C)** Validation of the NeuroD1 regulation on six ferroptosis-related genes predicted as targets of NeuroD1 using qRT-PCR in HCC-LM3. **(D–E)** NeuroD1 and GPX4 mRNA (D) and protein (E) expression levels in *NeuroD1*-knocked down HCC cells, as determined using qRT-PCR and western blotting, respectively. **(F–G)** NeuroD1 and GPX4 mRNA (F) and protein (G) expression levels in *NeuroD1*-overexpressed HCC cells, as determined using qRT-PCR and western blotting, respectively. **(H)** NeuroD1 and GPX4 protein expression levels in *NeuroD1*-overexpressed HCC-LM3 cells treated with 5 μM RSL-3 for 24 h, as determined using western blotting. Cells transfected with shCon or pcCon were used as controls. β-actin was used for qRT-PCR normalization and as western blotting loading control. Quantification data are expressed as mean ± SD (n = 3). *P* values were calculated using two-tailed unpaired Student’s *t*-test. shND1: shRNA expression vector targeting *NeuroD1*; pcCon: pcEF9-Puro; pcND1: *NeuroD1* overexpression vector; *P < 0.05; **P < 0.01; NS: not significant.

NeuroD1 forms a basic helix-loop-helix structure [33]; furthermore, it could form a complex with RNA polymerase II in HCC-LM3 cells (Fig 3A), thereby confirming its function as a transcription factor in HCC cells. To elucidate the molecular mechanism of its regulation of ferroptosis, we performed bioinformatics analysis by crossing a ChIP-sequencing dataset containing DNA fragments that could be bound by NeuroD1 (https://www.ncbi.nlm.nih.gov/geo/query/acc.cgi?acc=GSE179072) with a signature of 40 ferroptosis-related genes, as reported previously [48, 49] (Fig 3B). Accordingly, we identified six ferroptosis-related genes as potential NeuroD1 target genes. Among them, NCOA4 is involved in iron homeostasis, while GPX4, NFE2L2, FDFT1, ALOX12, and LPCAT3 are involved in lipid peroxidase reduction [44]. Next, we investigated their mRNA expression levels in *NeuroD1*-knocked down HCC-LM3 cells. As shown in Fig 3C, *NeuroD1* significantly altered the expression levels of GPX4 in HCC-LM3 cells. GPX4 is a selenoprotein that reduces lipid peroxidation, and thereby suppresses ferroptosis, by functioning as a GSH-dependent peroxidase [50]. These results indicate that NeuroD1 may regulate lipid peroxidase reduction in HCC cells.

Next, we verified the positive effect of NeuroD1 on GPX4 expression. Knocking down *NeuroD1* significantly suppressed GPX4 expression at both the mRNA and protein levels in HCC-LM3 cells (Fig 3D and 3E). Consequently, overexpressing *NeuroD1* robustly upregulated these genes (Fig 3F and 3G). To confirm this, we examined the effect of overexpressing *NeuroD1* on HCC-LM3 cells treated with RSL-3, a GPX4 inhibitor. RSL-3 significantly suppressed GPX4 expression, while *NeuroD1* overexpression clearly restored it (Fig 3H).

**Fig 4:**
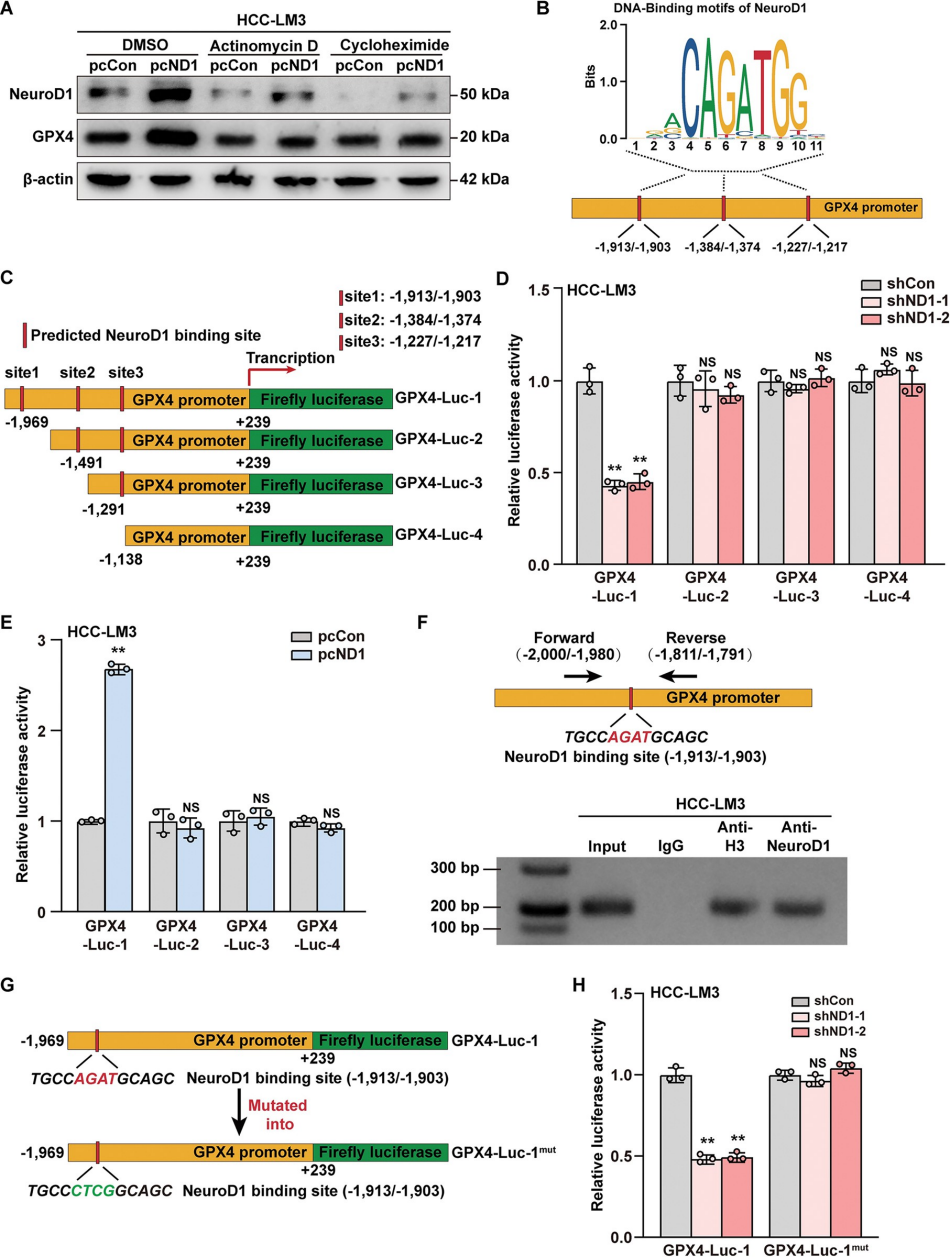
NeuroD1 directly binds to the *GPX4* promoter and regulates its transcriptional activity. **(A)** NeuroD1 and GPX4 protein expression levels in *NeuroD1*-overexpressed HCC-LM3 cells treated with actinomycin D or cycloheximide, as determined using western blotting. **(B)** Schematic diagram of the predicted NeuroD1 binding sites on the *GPX4* promoter. **(C)** Schematic diagram of *GPX4* reporter vectors (GPX4-Lucs). **(D–E)** Relative luciferase activities of GPX4-Luc-1 to GPX4-Luc-4 in *NeuroD1*-knocked down (D) and *NeuroD1*-overexpressed (E) HCC-LM3 cells. **(F)** Binding capacity of NeuroD1 to the predicted region on the *GPX4* promoter in HCC-LM3 cells, as examined using ChIP assay with anti-NeuroD1 antibody followed by PCR. Predicted NeuroD1 binding site on the *GPX4* promoter and the location of the primer set used for PCR are shown. Anti-histone H3 antibody was used as a positive control. **(G)** Schematic diagram of NeuroD1 binding site-mutated *GPX4* reporter vector (GPX4-Luc-1^mut^). Mutated base pairs are indicated in red and green. (**H)** Relative activity of GPX4-Luc-1^mut^ in *NeuroD1*-knocked down HCC-LM3 cells. Cells transfected with shCon or pcCon were used as controls. Quantification data are expressed as mean ± SD (n = 3). *P* values were calculated using two-tailed unpaired Student’s *t*-test. shND1: shRNA expression vector targeting *NeuroD1*; pcCon: pcEF9-Puro; pcND1: *NeuroD1* overexpression vector; **P < 0.01; NS: not significant.

To unravel the molecular mechanism underlying NeuroD1 regulation of GPX4, we examined the effect of inhibiting *de novo* transcription and protein synthesis in *NeuroD1*-knocked down HCC-LM3 cells. While knocking down *NeuroD1* greatly suppressed the expression of GPX4, inhibition of both transcription and *de novo* protein synthesis abolished this effect (Fig 4A), suggesting that NeuroD1 regulation of GPX4 expression occurs at its transcriptional stage. Next, to reveal whether NeuroD1 could directly bind to *GPX4* promoter and promote transcriptional activity, we first predicted possible NeuroD1 binding sites on the *GPX4* promoter. Using JASPAR (http://jaspar.genereg.net) [51], we identified three potential binding sites for the NeuroD1 core binding sequence, CAGATG, at positions -1,913 to -1,903; -1,384 to -1,374; and -1,227 to -1,217 of the *GPX4* promoter (Fig 4B). Based on this prediction, we constructed a series of luciferase reporter vectors carrying the -1,969 to +239, -1,491 to +239, -1,291 to +239, and -1,138 to +239 fragments of the *GPX4* promoter (Fig 4C). Luciferase reporter assay results showed that knocking down *NeuroD1* significantly suppressed the activity of GPX4-Luc-1 in HCC-LM3 and MHCC-97H cells; however, it failed to affect the activities of GPX4-Luc-2, GPX4-Luc-3, and GPX4-Luc-4 (Figs 4D and S6A). Similarly, *NeuroD1* overexpression robustly increased the activity of GPX4-Luc-1 without significantly affecting other GPX4 luciferase reporter vectors (Figs 4E and S6B). These results indicate that NeuroD1 upregulates *GPX4* transcriptional activity, and that the -1,969 to -1,492 region of the *GPX4* promoter is crucial for this regulation.

To analyze whether NeuroD1 could bind to the *GPX4* promoter, especially to the fragment containing the -1,913 to -1,903 region of the *GPX4* promoter, we performed a ChIP assay with a primer set flanking it. The results demonstrated that NeuroD1 could directly bind to the -2,000 to -1,791 region of the *GPX4* promoter in HCC-LM3 and MHCC-97H cells (Figs 4F and S6C). Finally, we constructed a mutant GPX4-Luc-1 reporter vector (GPX4-Luc-1^mut^) by mutating the AGAT sequence in the predicted NeuroD1 binding site on the -1,913 to -1,903 region of the *GPX4* promoter to CTCG (Fig 4G). Our results showed that, unlike its effect on GPX4-Luc-1, knocking down *NeuroD1* failed to exert any significant effect on GPX4-Luc-1^mut^ (Fig 4H). Taken together, our results indicate that NeuroD1 regulates the transcriptional activity of the *GPX4* promoter, most plausibly through direct binding to the -1,913 to -1,903 region.

### NeuroD1/GPX4 is crucial for HCC ferroptosis resistance

**Fig 5:**
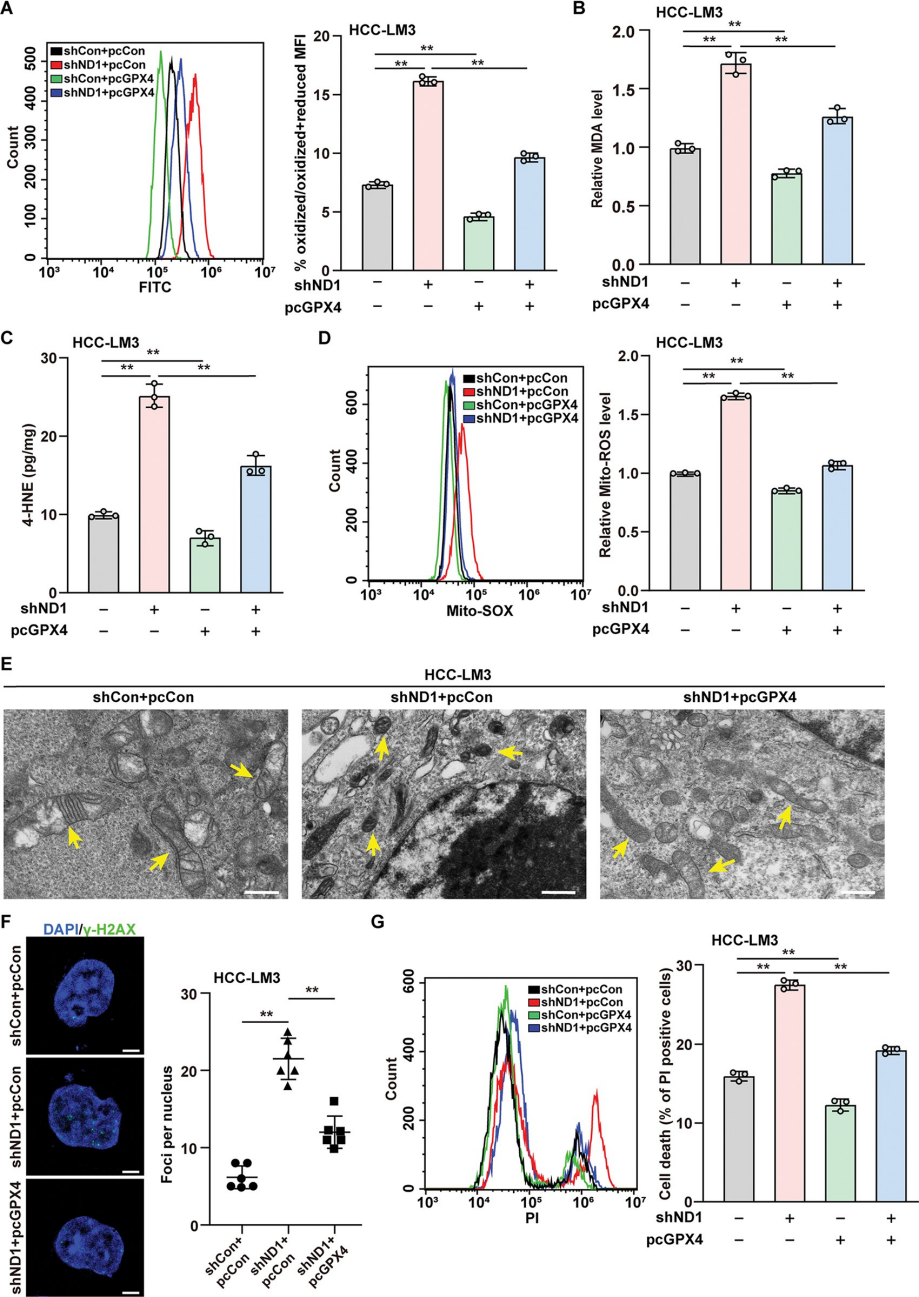
NeuroD1 suppresses ferroptosis by upregulating GPX4. **(A)** Lipid peroxidation level in *NeuroD1*-knocked down, *GPX4*-overexpressed HCC-LM3 cells, as assessed using flow cytometry. Data were presented as % of oxidized/oxidized + reduced MFI (*F* value = 1762). **(B–C)** MDA (B; *F* value = 138) and 4-HNE (C; *F* value = 166.2) levels in *NeuroD1*-knocked down, *GPX4*-overexpressed HCC-LM3 cells. **(D)** Mitochondrial ROS level in *NeuroD1*-knocked down, *GPX4*-overexpressed HCC-LM3 cells, as assessed by Mito-SOX staining and flow cytometry (*F* value = 939.1). **(E)** Mitochondrial morphology in *NeuroD1*-knocked down, *GPX4*-overexpressed HCC-LM3 cells, as examined using transmission electron microscope. Yellow arrows: mitochondria. Scale bars: 1 μm. **(F)** γ-H2AX expression level in *NeuroD1*-knocked down, *GPX4*-overexpressed HCC-LM3 cells, as analyzed using immunofluorescent staining. Representative images (left; scale bars: 5 μm) and quantification results (right; n = 6; *F* value = 78.89) are shown. **(G)** Cell death rate in *NeuroD1*-knocked down, *GPX4*-overexpressed HCC-LM3 cells, as examined using PI staining and flow cytometry (*F* value = 377.8). Cells transfected with shCon and/or pcCon were used as controls. Total protein was used for normalizing MDA and 4-HNE levels. Quantification data are expressed as mean ± SD (n = 3; unless otherwise indicated). One-way ANOVA and Tukey multiple comparisons analyses were performed when more than two groups were compared. shND1: shRNA expression vector targeting *NeuroD1*; pcCon: pcEF9-Puro; **P < 0.01.

To unravel the role of GPX4 in NeuroD1-mediated ferroptosis, we constructed a *GPX4* overexpression vector (S7A Fig) and restored the level of *GPX4* suppressed by knocking down *NeuroD1* in HCC-LM3 cells (S7B Fig). Concomitantly, overexpression of *GPX4* canceled the increase in lipid ROS, MDA, and 4-HNE levels (Fig 5A–5C) mediated by *NeuroD1* knockdown in HCC-LM3 cells, indicating that GPX4 abolished the increase in lipid peroxidation triggered by knocking down *NeuroD1*. Accordingly, in HCC-LM3 cells, *GPX4* overexpression suppressed the increase in mitochondrial ROS and restored mitochondrial shrinkage induced by knocking down *NeuroD1* (Fig 5D and 5E). Furthermore, *GPX4* overexpression abolished the effect of *NeuroD1* knockdown on promoting DNA damage and the percentage of HCC-LM3 cell death (Fig 5F and 5G), leading to an increase in HCC-LM3 cell viability and colony formation potential (S7C and S7D Fig). Moreover, the inhibition of GPX4 expression by RSL-3 treatment robustly restored lipid ROS levels, and the percentage of PI-positive cells, which were suppressed by *NeuroD1* overexpression in HCC-LM3 cells (S8A and S8B Fig). Meanwhile, *NeuroD1* overexpression resulted in significantly higher viability at every RSL-3 concentration tested (S8C Fig). These results demonstrate that NeuroD1/GPX4 is crucial for ferroptosis resistance in HCC cells.

### NeuroD1/GPX4 axis-mediated ferroptosis resistance is crucial for promoting hepatocarcinogenesis

**Fig 6:**
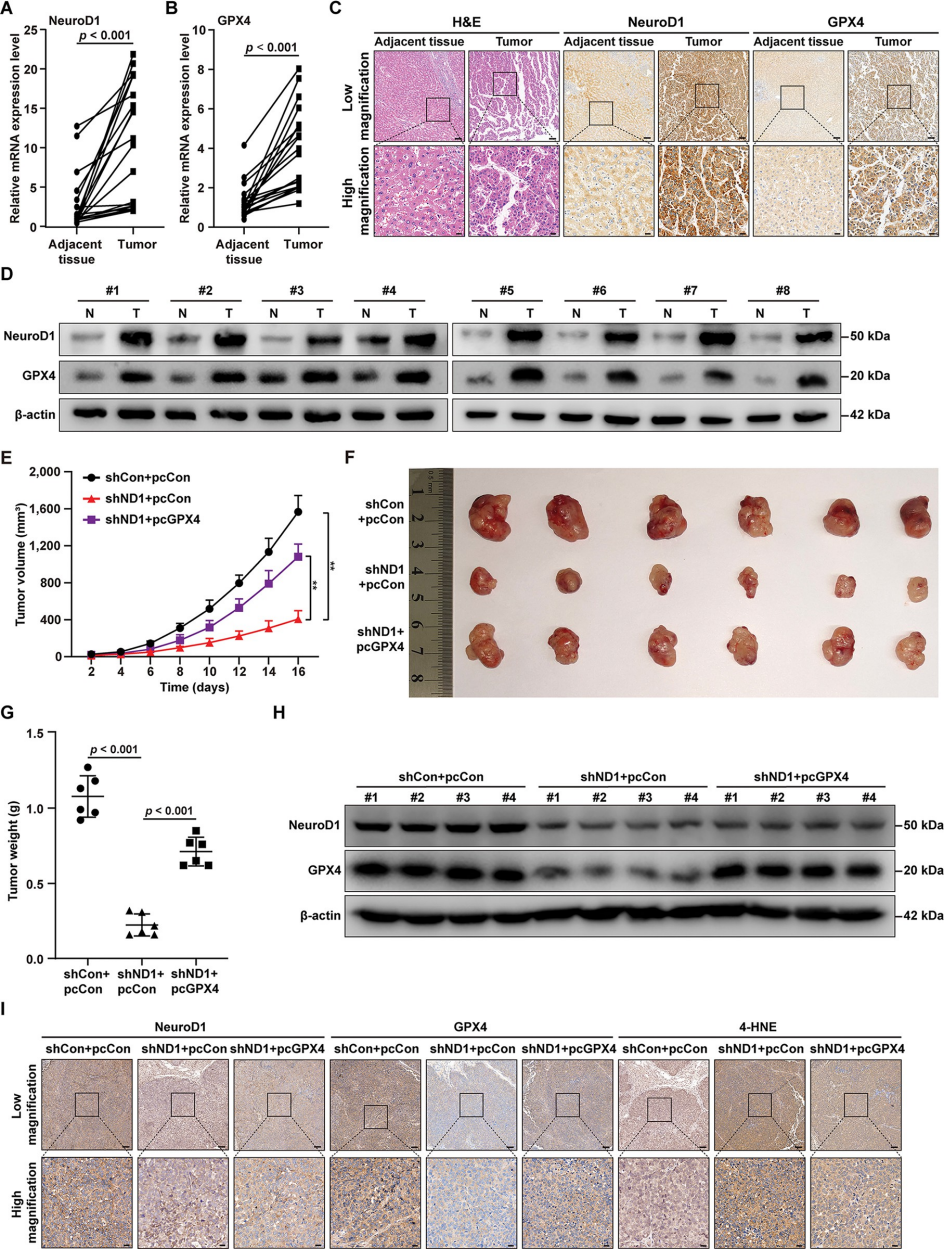
NeuroD1/GPX4 axis promotes HCC cell ferroptosis resistance and tumorigenic potential. **(A–B)** NeuroD1 (A) and GPX4 (B) mRNA expression levels in clinical HCC and corresponding normal adjacent tissues, as analyzed using qRT-PCR (n = 20). **(C–D)** NeuroD1 and GPX4 protein expression levels in clinical HCC and corresponding normal adjacent tissues, as analyzed using immunohistochemical staining (C; top panels: low magnification, scale bars:100 μm; bottom panels: high magnification, scale bars: 20 μm.) and western blotting (D; n = 8). **(E–G)** Tumorigenic potentials of HCC-LM3/shCon+pcCon, HCC-LM3/shND1+pcCon, and HCC-LM3/shND1+pcGPX4 stable cell lines, as examined *in vivo* by subcutaneous injection of these cells into Balb/c-nu/nu mice (n = 6). Tumor volumes at indicated time points (E; *F* value = 102.4), morphological images of the tumors generated (F), and tumor weight at day 16 after transplantation (G; *F* value = 98.65) are shown. **(H)** NeuroD1 and GPX4 protein expression levels in the xenografted tumors formed by the indicated cells, as determined using western blotting. **(I)** NeuroD1, GPX4, and 4-HNE expression levels in the xenografted tumors formed by indicated cell lines, as determined using immunohistochemistry staining (top panels: low magnification, scale bars:100 μm; bottom panels: high magnification, scale bars: 20 μm). β-actin was used for qRT-PCR normalization and as western blotting loading control. Quantification data are expressed as mean ± SD. *P* values were calculated using two-tailed unpaired Student’s *t*-test, or using one-way ANOVA and Tukey multiple comparisons when more than two groups were compared. shND1: shRNA expression vector targeting *NeuroD1*; pcCon: pcEF9-Puro; **P < 0.01.

To confirm the role of the NeuroD1/GPX4 axis in promoting hepatocarcinogenesis, we first analyzed their expression in HCC tissues and corresponding normal adjacent tissues. Both NeuroD1 and GPX4 mRNAs were expressed at significantly higher levels in HCC tissues than in normal adjacent tissues (Fig 6A and 6B). This tendency was further confirmed at the protein level, as shown by immunohistochemistry and western blotting (Fig 6C and 6D). Furthermore, clinical data from TCGA and GTEx further confirmed the increase of NeuroD1 and GPX4 in various tumor tissues (S9A and S9B Fig). Together, these results suggested a positive correlation between NeuroD1 and GPX4 in tumor tissues, further confirming the possible regulation of NeuroD1 on GPX4 as obtained by cellular experiments.

Finally, to examine the role of NeuroD1/GPX4 in promoting hepatocarcinogenesis *in vivo*, we performed xenograft experiments using *NeuroD1*-knocked down as well as *NeuroD1*-knocked down/*GPX4*-overexpressed HCC-LM3 stable cell lines (S10 Fig). Knocking down *NeuroD1* clearly suppressed the tumor growth rate, as shown by the changes in tumor volume, while overexpressing *GPX4* restored it (Fig 6E). The same tendency was also confirmed by tumor morphology, which showed significantly smaller tumors formed by *NeuroD1*-knocked down HCC-LM3 cells and a clear restoration effect by overexpression of *GPX4* (Fig 6F), as well as by tumor weight (Fig 6G). Western blotting (Fig 6H) and immunohistochemical staining (Fig 6I) confirmed that GPX4 expression was downregulated in the tumor lesions formed by *NeuroD1*-knocked down HCC-LM3 cells. Furthermore, through immunohistochemical staining using 4-HNE, we confirmed *in vivo* that *NeuroD1* knockdown robustly increased the level of lipid peroxidase, whereas overexpression of *GPX4* suppressed it (Fig 6I).

**Fig 7:**
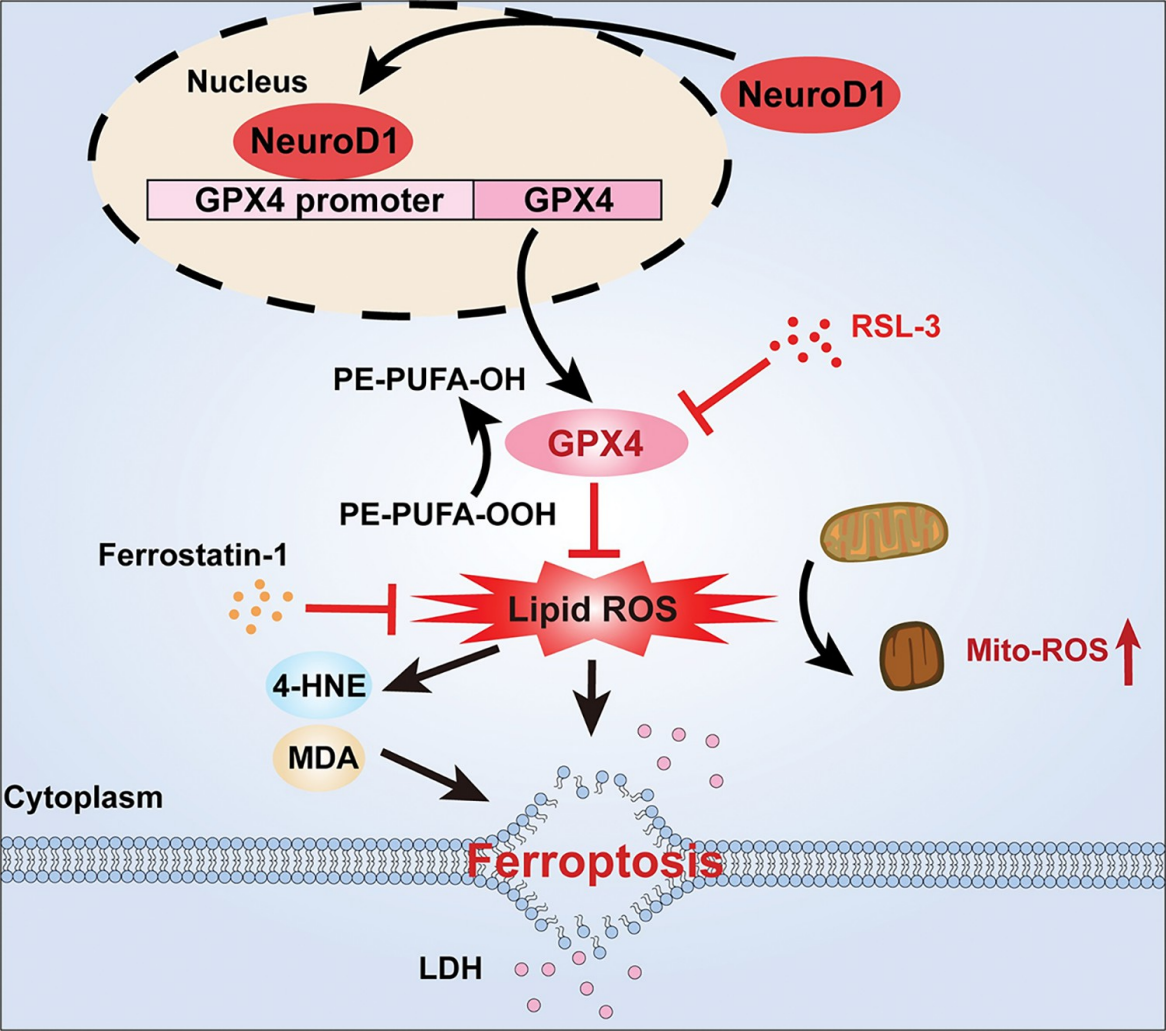
Schematic diagram showing the role of NeuroD1/GPX4 axis on HCC cell ferroptosis resistance and tumorigenesis.

In summary, our results reveal that NeuroD1 regulates HCC cells death resistance by suppressing ferroptosis, and that this regulation occurs through the function of NeuroD1 as a transcription factor that binds to the *GPX4* promoter and enhances its transcriptional activity (Fig 7).

## Discussion

Cell death is a fundamental physiological process occurring in almost all human cells. Different types of cell death such as apoptosis, necroptosis, and ferroptosis trigger different cellular reactions and affect disease progression via distinct mechanisms [52]. Ferroptosis is an iron-dependent cell death process mediated by lipid peroxides. The main mechanism of ferroptosis involves bivalent iron and lipoxygenase, which catalyzes the peroxidation of PUFAs in the cell membrane, leading to cell death [53]. Aberrant iron metabolism leads to the Fenton reaction and enhances oxidative stress, resulting in ROS accumulation and lipid peroxidation. This, in turn, damages the mitochondria, causing shrunken mitochondria with decreased crista, condensed membrane, and ruptured outer membrane [50]. Furthermore, disruption of redox homeostasis due to aberrant amino acid metabolism, such as glutamate-cysteine exchange and GSH synthesis, and by the impaired GPX4 expression, leads to defects in the ability to reduce lipid peroxidation, and thus is a crucial factor that triggers ferroptosis [54]. Meanwhile, an increase in ferroptosis resistance has been observed in various tumors, including HCC as well as esophageal, gastric, colorectal, and breast cancers [55–59]; thus, inducing ferroptosis has attracted attention as a potential strategy to overcome tumor drug resistance [60]. In this study, we identified NeuroD1 as a regulator of HCC cell death, leading to an increase in cell viability, and subsequently, tumorigenic capacity. Following inhibitor treatment, we found that *NeuroD1* knockdown triggered apoptosis and ferroptosis in HCC cells, with ferroptosis contributing the most. Hence, this study revealed a novel regulatory mechanism of ferroptosis resistance in tumor cells. Furthermore, given that iron overload and oxidative stress are the two major factors that trigger liver injury, ferroptosis has been reported to be involved in multiple liver diseases [61]. Therefore, although further investigations are required, this study suggests that NeuroD1/GPX4 regulation may also be involved in other liver diseases.

Lipid peroxidation is a characteristic feature of ferroptosis. GPX4 is a key protein in the cellular response to oxidative stress and in maintaining cellular lipid homeostasis as it can specifically catalyze GSH-mediated conversion of lipid peroxides into lipid alcohols [62, 63]. Defects in GSH and GPX4 lead to the accumulation of toxic lipid peroxides in cells, eventually leading to ferroptosis [64]. GPX4 expression is upregulated in various tumors and is closely related to tumorigenesis, metastasis, and poor prognosis [62, 65]. Previous studies have shown that GPX4 promotes the growth of glioblastomas as well as the migration and invasion potential of gastric and renal cancers [66, 67]. In this study, we showed that NeuroD1 could bind directly to the *GPX4* promoter, thereby upregulating its transcriptional activity. This, in turn, promotes the ability of HCC to reduce lipid peroxidase and thus suppresses ferroptosis while promoting viability. However, while the effect of knocking down *NeuroD1* with shRNA expression vectors in HCC cells was significant, it is noteworthy that the expression of NeuroD1 was not totally abolished; hence, it is plausible that NeuroD1 also regulate other ferroptosis-related factors, although less significant compared to its regulation on GPX4. Nevertheless, our study clearly showed for the first time that NeuroD1 is involved in ferroptosis resistance in HCC cells.

Tumor cells exhibit vigorous metabolism and unlimited cell proliferation, resulting in an increased iron demand and higher levels of oxidative stress. To survive, tumor cells must generate high concentrations of antioxidants to mitigate oxidative stress [68]. Given that ferroptosis is iron- and ROS-dependent, tumor cells are more susceptible to ferroptosis [16]. Increasing evidence suggests that tumor cells with high resistance to antitumor therapeutic strategies, such as radiotherapy, chemotherapy, targeted therapy, and immunotherapy, respond better to ferroptosis; thus, inducing ferroptosis has been considered a potential strategy for enhancing the efficacy of these therapeutic strategies [69, 70]. Furthermore, previous studies have shown that tumor cells in specific cellular states are susceptible to ferroptosis [71, 72]. For example, during the epithelial-mesenchymal transition (EMT), mesenchymal tumor cells are typically resistant to conventional therapy-induced apoptosis. However, they are more sensitive to ferroptosis due to an increase in PUFA synthesis and a strong reliance on GPX4 to detoxify lipid peroxides [30, 73]. Our study showed that NeuroD1 could promote the expression of GPX4, thereby enhancing cell antioxidant capacity and preventing harmful oxidative stress caused by the rapid proliferation of tumor cells. This in turn enhances the ferroptosis resistance, and subsequently promotes HCC progression. Hence, targeting NeuroD1-mediated ferroptosis resistance may be a potential strategy for sensitizing tumor cells to chemotherapy and radiotherapy. However, it is noteworthy that NeuroD1 is also related with other diseases, as inactivation of NeuroD1 could triggers a downregulation of endocrine differentiation transcription factors and upregulation of non-endocrine genes, thereby disturbing endocrine balance [74]. Moreover, mutation in *NeuroD1* could lead to the defect in pancreatic B cells and thus promote type 2 diabetes mellitus (T2DM), while patients with NeuroD1 Thr45 polymorphism are more susceptible to T2DM [75, 76]. Therefore, a targeted therapy specific to NeuroD1, or intratumoral administration of NeuroD1 inhibitor might be considered to avoid the side-effects of antitumor therapeutic strategy targeting NeuroD1.

Previous studies have shown that NeuroD1, originally discovered as a factor regulating neuronal development, is upregulated in several types of cancer, including neuroblastoma, small cell lung cancer, colon cancer, breast cancer, and pancreatic cancer [41,42,77–80]. Furthermore, recent studies have shown that it promotes the migration and invasion of neuroendocrine carcinoma and pancreatic cancer cells [39, 81]. Our previous study also revealed that NeuroD1 could promote tumor cell metabolic reprogramming, as it could enhance the pentose phosphate pathway (PPP) by promoting the transcription of the PPP rate-limiting enzyme G6PD [42]. In this study, we uncovered a novel oncogenic function of NeuroD1 as a suppressor of ferroptosis in tumor cells, thereby establishing a link between NeuroD1 and cell death resistance, which is a major hallmark of cancer. To our knowledge, this is the first study to demonstrate the role of NeuroD1 in promoting HCC, further emphasizing its significant role as a driving force of tumor progression.

In summary, our study revealed a novel function of NeuroD1 in the transcriptional regulation of GPX4 and demonstrated its critical role in tumor cell ferroptosis resistance through direct binding to *GPX4* promoter and activation of its transcriptional activity. Our study elucidates the underlying mechanism of ferroptosis in tumor cells, providing a promising novel approach for future therapies by targeting the NeuroD1/GPX4 axis.

## Material and methods

### Ethics statement

Animal study was carried out in the Chongqing University Cancer Hospital, and was approved by the Laboratory Animal Welfare and Ethics Committee of Chongqing University Cancer Hospital. All animal experiments conformed to the approved guidelines of the Animal Care and Use Committee of the Chongqing University Cancer Hospital. All efforts were made to minimize suffering.

For the clinical HCC samples, prior patients’ written informed consents were obtained. The study was approved by the Institutional Research Ethics Committee of Chongqing University Cancer Hospital, and conducted in accordance with Declaration of Helsinki.

### Vector constructions

shRNA control vector (shCon), shRNA expression vectors targeting *NeuroD1*, as well as *NeuroD1* overexpression vectors were constructed as described previously [41]. Target sequences specific for *NeuroD1* are as follow: shNeuroD1-1: 5′-GCACAAGCTTGTATATACA-3′, and shNeuroD1-2: 5′-GCTGCAAAGTGCAAATAC-3′. For *GPX4* overexpression vector (pcGPX4), the coding region of human *GPX4* was amplified using the Takara Ex Taq Kit (Takara Bio, Dalian, China) from human cDNA obtained by reverse-transcribing total RNA extracted from HCC-LM3 WT cells using the PrimeScript RT Reagent Kit with gDNA Eraser (Takara Bio). The amplicon was inserted into the *Bam*HI and *Eco*RI sites of pcEF9-Puro and brought puromycin resistance gene [82].

For GPX4 luciferase reporter vectors, the -1,969 to +239 (GPX4-Luc-1), -1,491 to +239 (GPX4-Luc-2), -1,291 to +239 (GPX4-Luc-3), and -1138 to +239 (GPX4-Luc-4) regions of the *GPX4* promoter were cloned into the *Xho*I and *Hind*III sites of the pGL4-SV40 vector (Promega, Madison, WI). Human genome DNA extracted from wild-type HCC-LM3 cells using TIANamp Genomic DNA Kit (Tiangen Biotech, Beijing, China) was used as a template for amplifying the promoter regions. GPX4-luciferase vector with mutated NeuroD1 binding site (GPX4-Luc-1^mut^) was constructed based on the site-specific mutagenesis method using a Site-directed Gene Mutagenesis Kit (Beyotime Biotechnology, Shanghai, China).

### Cell lines and cell culture

HCC HCC-LM3 and MHCC-97H cells were purchased from the Cell Bank of Chinese Academy of Sciences (Shanghai, China), and cultured in Dulbecco’s modified Eagle’s medium (Gibco, Life Technologies, Grand Island, NY) with 10% FBS (Biological Industries, Beith Haemek, Israel) and 1% penicillin–streptomycin. Cell lines were verified using short-tandem repeat profiling method, and were tested for mycoplasma contamination by using Mycoplasma Detection Kit-QuickTest (Biotool, Houston, TX) routinely every 6 months. Cells were transfected with indicated vectors using Lipofectamine 2000 (Invitrogen Life Technologies, Carlsbad, CA) according to the manufacturer’s protocol.

For knockdown and overexpression experiments, cells were seeded in 6-well plates before being transfected with 2 μg of indicated shRNA expression and overexpression vectors. Twenty-four hours after transfection, puromycin selection was performed to eliminate untransfected cells. For the rescue experiments, cells were seeded in 6-well plates before being transfected with 1 μg of shNeuroD1 and 1 μg pcGPX4 vector and subjected to puromycin selection.

For establishing *NeuroD1*-knocked down HCC-LM3 cells (HCC-LM3/shNeuroD1 cells) and *NeuroD1*-knocked down, *GPX4*-overexpressed HCC-LM3 cells (HCC-LM3/shNeuroD1/pcGPX4 cells), cells were seeded in 10 cm well plates, and transfected with 12 μg of shCon or shNeuroD1 and 6 μg of pcCon or pcGPX4 vectors. Stable cell lines were established by performing puromycin selection.

For experiments treated with Z-VAD (APExBIO, Houston, United States), NSA (MedChem Express, New Jersey, United States), 3-MA (MedChem Express), Ferr-1 (MedChem Express), RSL-3 (Macklin Biochemical, Shanghai, China) and NAC (MedChem Express), treating cells with indicated concentrations for indicated times before detection.

### Clinical HCC samples

Clinical HCC samples were obtained from HCC patients undergoing surgery at Chongqing University Cancer Hospital. Patients did not receive chemotherapy, radiotherapy or other adjuvant therapies prior to the surgery. The specimens were snap-frozen in liquid nitrogen.

### Animal experiment

For the *in vivo* tumor study, BALB/c-nu/nu mice (male; body weight, 18–22 g; 6 weeks old) were purchased from Chongqing Medical University. To generate an experimental subcutaneous tumor model, BALB/c-nu/nu mice were randomly divided into three groups (n = 6), and each group was injected subcutaneously with 3 × 10^6^ of indicated stable cell lines. Tumor size (V) was evaluated by a caliper every 2 days with reference to the following equation: V = a × b^2^ /2, where a and b are the major and minor axes of the tumor, respectively. The investigator was blinded to the group allocation and during the assessment.

### ROS and lipid peroxidation

Cells were prepared as described above. ROS was detected with DCFH-DA fluorescent probe using Reactive Oxygen Species Assay Kit (Beyotime Biotechnology) according to the manufacturer’s instruction. Lipid peroxidation was detected using C11-BODIPY (Invitrogen Life Technologies) to assess % of oxidized/oxidized + reduced MFI according to the manufacturer’s protocol.

### MDA and 4-HNE

Cells were prepared as described above. The levels of MDA and 4-HNE were detected by Lipid Peroxidation MDA Assay Kit (Beyotime Biotechnology) and human 4-HNE ELISA Kit (Fine Test, Wuhan, China), according to the manufacturers’ protocols.

### Mitochondrial ROS

Cells were prepared as described above. Mitochondrial ROS was analyzed by Mito-Sox Red Mitochondrial Superoxide Indicator (Yeasen, Shanghai, China) and flow cytometry according to the manufacturer’s guidelines.

### Transmission electron microscopy

Cells were prepared as described above and fixed with 2.5% glutaraldehyde, washed in 0.1M phosphate buffer (pH 7.4), and post-fixed with 1% osmium tetroxide, dehydrated with serial acetone, infiltrated, and embedded in Epox 812. After ultra-thin sectioning, the sections were stained with uranyl acetate and lead citrate, and then examined with a Jem-1400-Flash transmission electron microscope (JEOL, Tokyo, Japan).

### Cell viability and cell death

Cells were prepared as described above. Cell viability was assessed by counting the total number of viable cells using MTS (Promega) according to the manufacturer’s protocol. To detect the cell death rate, cells were stained with propidium iodide (PI, Neobioscience, Beijing, China), and subjected to flow cytometry.

### Time-lapse microscopy

For live-imaging of cell death, cells were prepared as described above and re-seed in 3.5-cm glass bottom dishes for live cell image system. (Wuxi NEST Biotechnology, Wuxi, China; 1 × 10^5^ cells/well cells/dish). The medium was changed to the medium containing PI (1:100) 30 min prior to imaging. Images were acquired every 10 min for 6 h with a 20-ms exposure time by fluorescence microscope Olympus IX83 (Olympus, Tokyo, Japan). The images were processed with FLUOVIEW (v.2.3). The cell death rate and morphology were analyzed.

### LDH release assay

Cells were prepared as described above, re-seeded into 24 wells dish (6 × 10^4^ cells/well), and cultured for 24 h. The supernatants were collected, and the level of the LDH released into the medium was measured using LDH Cytotoxicity Assay Kit (Beyotime Biotechnology), according to the manufacturer’s protocol.

### Dual luciferase reporter assay

HCC-LM3 cells were seeded into 24-well plates at a density of 6 × 10^4^ cells/well and co-transfected with indicated shRNA expression vectors or overexpression vectors, reporter vectors bringing the firefly luciferase, and *Renilla* luciferase expression vector (pRL-SV40, Promega) as internal control. After 24 h, luciferase activities were detected using the Dual Luciferase Reporter Assay (Promega). The activities of *Renilla* luciferase were used to normalize those of the firefly luciferase from the corresponding samples. The results were shown as relative to the expression level in the corresponding controls, which were assumed as 1.

### RNA extraction and quantitative real-time PCR (qRT-PCR)

Cells were prepared as described above. Total RNA was extracted with Trizol (Invitrogen Life Technologies) according to the manufacturer’s instruction. Total RNA (1 μg) was reverse-transcribed into cDNA using the PrimeScript RT Reagent Kit with gDNA Eraser (Takara Bio), and qRT-PCR was performed using SYBR Premix Ex Taq (Takara Bio) to assess mRNA expression levels. β-actin was used to normalize sample amplifications. Primer sequences used were listed in S1 Table. Results were shown as the mRNA levels relative to the corresponding control, which was assumed as 1.

### Immunofluorescent staining

Cells were prepared as described above and re-seeded into 24-well plates at a density of 6 × 10^4^ cells/well. Twenty-four hours later, cells were fixed with 4% paraformaldehyde for 15 min and permeabilized with PBS containing 1% Triton X-100 for 10 min. Blocking was performed with 5% BSA. Cells were then incubated with primary antibodies for 2 h before being incubated further with secondary antibodies. Nuclei were stained with DAPI (Beyotime Biotechnology). Images were taken and analyzed with laser scanning confocal microscopy (Leica Microsystems TCS SP5, Leica, Heidelberg, Germany). Antibodies used were listed in S2 Table.

### Immunohistochemistry and Hematoxylin-Eosin (H&E) staining

Fresh human HCC tissues, normal adjacent tissues, and xenografted tumors were fixed using 4% paraformaldehyde for overnight prior to being embedded in paraffin and sectioned at 4 μm thickness using a cryostat. Sections were then dewaxed using xylene, rehydrated, and subjected to immunohistochemistry. Briefly, the tissue sections were incubated with primary antibodies for 1 h before being incubated with corresponding secondary antibodies conjugated with horse-radish peroxidase. Visualization was performed using a DAB Kit (DAKO, Beijing, China) under microscope. The nuclei were then counterstained with hematoxylin, followed by dehydration and coverslip mounting. Images were obtained using Pannoramic MIDI (3DHistech, Budapest, Hungary). The antibodies used were listed in S2 Table.

For hematoxylin-eosin (H&E) staining, paraffin sections from human HCC tissues and normal adjacent tissues (4 μm thickness) were fixed in 10% formalin and washed with 60% propylene glycerol. The samples were then stained with 0.5% hematoxylin-eosin (Sangon Bio, Shanghai, China) for 3 min followed by dehydration and coverslip mounting. Images were taken using Pannoramic MIDI (3DHistech).

### Immunoprecipitation assay

Cells were seeded in a 10 cm dish (5 × 10^6^ cells per dish), and transfected with 16 μg NeuroD1 overexpressing vectors. Total protein samples were collected and lysed with RIPA lysis buffer with protease inhibitor and phosphatase inhibitor cocktail (complete cocktail, Roche Applied Science, Mannheim, Germany), and cleared by centrifugation at 12,000 rpm. The supernatants were incubated at 4°C for 4 h with protein A+G beads (Beyotime Biotechnology) in the presence of indicated antibodies or IgG as control. The immunoprecipitated proteins were then subjected to immunoblotting analysis as described in western blotting. The antibodies used were listed in S2 Table.

### Chromatin immunoprecipitation (ChIP) assay

Chromatin was immunoprecipitated using the ChIP Assay Kit (Beyotime Biotechnology) according to the manufacturer’s instruction. Briefly, cells were lysed and chromatins were immunoprecipitated using protein A+G Agarose/salmon sperm DNA and anti-NeuroD1 antibody, anti-H3 antibody, or normal rabbit IgG, de-crosslinked for 4 h at 65°C, and treated with 0.5 M EDTA, 1 M Tris (pH 6.5), and 20 mg/mL proteinase K. Immunoprecipitated chromatin was then subjected to PCR by using PrimeSTAR Max (Takara Bio). Primer sequences used for amplifying the *GPX4* promoter region with the predicted NeuroD1 binding site were: 5’-CTTATGCAAGACCAGGATTCG-3’ (forward); and 5’-TCTTAACCTTTTCTGACCCTG -3’ (reverse). The antibodies used were listed in S2 Table.

### Western blotting

Cells were prepared as described above before being lysed using RIPA lysis buffer with protease inhibitor and phosphatase inhibitor cocktail (complete cocktail; Roche Applied Science). For clinical tissues and xenografted tumors, proteins were extracted from the frozen specimens using RIPA lysis buffer with protease inhibitor and phosphatase inhibitor cocktail. Equal amounts of proteins (20 μg) were electrophoresed on sodium dodecyl sulfate polyacrylamide gel and transferred to a polyvinylidene fluoride (PVDF) membrane with 0.45 μm pores (Millipore, Billerica, MA). The signals were measured using SuperSignal West Femto Maximum Sensitivity Substrate detection system (Thermo Scientific, Waltham, MA). Antibodies used were listed in S2 Table. β-actin was used to normalize sample amplifications.

### Colony formation assay

Cells were prepared as described above before being re-seeded into 6-well plates at a density of 300 cells/well and cultured for 10 days. Cells were then fixed with 4% paraformaldehyde and stained with methylene blue. Quantification was performed by counting the number of the colonies formed. Investigator was blinded during the assessment.

### Human tumor RNA-sequencing analysis

RNA-sequencing data regarding expression levels for NeuroD1 and GPX4 from human tumors and matched normal tissues collected by TCGA, TARGET, and the Genotype Tissue Expression Project (GTEx) were downloaded as log_2_ (RNAseq-RESM expected counts+1) values from UCSC Xena (https://xenabrowser.net/) with the query “TCGA TARGET GTEx”. Expression levels of NeuroD1 and GPX4 in tumor and normal tissues were analyzed and presented as log_2_ (RNAseq-RESM expected_count+1). The data listed in SI Data.

### Statistical analysis

All the quantification results were presented as mean ± SD (n = 3, unless otherwise indicated). Statistical analysis was performed using two-tailed unpaired Student’s t-test conducted using GraphPad Prism 8.4. When more than two groups were compared, one-way ANOVA was performed first, then a Tukey multiple comparisons test was performed. A value of *P < 0.05 was considered statistically significant.

## Supporting information

S1 Fig Efficacies of shRNA expression vectors targeting *NeuroD1* and *NeuroD1* overexpression vector. **(A)** NeuroD1 mRNA expression level in HCC-LM3 cells transfected with two shRNA expression vectors targeting different sites of *NeuroD1*, as determined using qRT-PCR. **(B–C)** NeuroD1 protein expression levels in HCC-LM3 and MHCC-97H cells transfected with two shRNA expression vectors targeting different sites of *NeuroD1* (B) and *NeuroD1* overexpression vector (C), as determined using western blotting. Cells transfected with shCon or pcCon were used as controls. β-actin was used for qRT-PCR normalization and as western blotting loading control. Quantification data are expressed as mean ± SD (n = 3). *P* values were calculated using two-tailed unpaired Student’s *t*-test. shND1: shRNA expression vector targeting *NeuroD1*; pcCon: pcEF9-Puro; pcND1: *NeuroD1* overexpression vector; **P < 0.01.
(TIF)

S2 Fig NeuroD1 enhances HCC cell viability and colony formation potential. **(A)** Viability of *NeuroD1*-knocked down MHCC-97Hcells. **(B–C)** Colony formation potential of *NeuroD1*-knocked down HCC-LM3 (B) and MHCC-97H (C) cells. Representative images (left) and quantification results (right) are shown. **(D–E)** Viability of *NeuroD1*-overexpressed HCC-LM3 (D) and MHCC-97H (E) cells. **(F–G)** Colony formation potential of *NeuroD1*-overexpressed HCC-LM3 (F) and MHCC-97H (G) cells. Representative images (left) and quantification results (right) are shown. Cells transfected with shCon or pcCon were used as controls. Quantification data are expressed as mean ± SD (n = 6). *P* values were calculated using two-tailed unpaired Student’s *t*-test. shND1: shRNA expression vector targeting *NeuroD1*; pcCon: pcEF9-Puro; pcND1: *NeuroD1* overexpression vector; **P < 0.01.
(TIF)

S3 Fig NeuroD1 inhibits HCC cell death. **(A)** Cell death rate of *NeuroD1*-knocked down MHCC-97H cells, as examined using PI staining and flow cytometry. **(B–C)** Cell death rate of *NeuroD1*-overexpressed HCC-LM3 cells (B) and MHCC-97H cells (C), as examined using PI staining and flow cytometry. **(D)** Total cellular ROS level in *NeuroD1*-overexpressed HCC-LM3 cells. **(E)** Cell death rate of *NeuroD1* re-expressed *NeuroD1*-knocked down HCC-LM3 cells, as examined using PI staining and flow cytometry (*F* value = 134.8). **(F)** Total cellular ROS level in *NeuroD1* re-expressed *NeuroD1*-knocked down HCC-LM3 cells, as assessed using DCFH-DA staining and flow cytometry (*F* value = 133.9). (**G**) Total cellular ROS level in *NeuroD1* knocked down, N-acetylcysteine (NAC)-treated HCC-LM3 cells, as assessed using DCFH-DA staining and flow cytometry (*F* value = 234.1). (**H**) Relative viability of *NeuroD1*-knocked down HCC-LM3 cells treated with Z-VAD (final concentration: 20 μM) for 48 h (n = 6; *F* value = 109.3). Cells transfected with shCon, pcCon, or treated with DMSO were used as controls. Quantification data are expressed as mean ± SD (n = 3). *P* values were calculated using two-tailed unpaired Student’s *t*-test, or using one-way ANOVA and Tukey multiple comparisons when more than two groups were compared. shND1: shRNA expression vector targeting *NeuroD1*; pcCon: pcEF9-Puro; pcND1: *NeuroD1* overexpression vector; *P < 0.05, **P < 0.01.
(TIF)

S4 Fig NeuroD1 suppresses lipid ROS level in HCC. **(A)** Lipid peroxidation level in *NeuroD1*-knocked down MHCC-97H cells, as assessed using flow cytometry. Data were presented as % of oxidized/oxidized + reduced MFI. **(B–C)** Lipid peroxidation level in *NeuroD1*-overexpressed HCC-LM3 (B) and MHCC-97H (C) cells, as assessed using flow cytometry. Data were presented as % of oxidized/oxidized + reduced MFI. **(D–E)** MDA (D) and 4-HNE (E) levels in *NeuroD1*-overexpressed HCC-LM3 cells. **(F)** Mitochondrial ROS level in *NeuroD1*-overexpressed HCC-LM3 cells, as assessed by Mito-SOX staining and flow cytometry. **(G)** Level of LDH released from *NeuroD1*-overexpressed HCC-LM3 cells. Cells transfected with shCon or pcCon were used as controls. Total protein was used for normalizing MDA and 4-HNE levels. Quantification data are expressed as mean ± SD (n = 3). *P* values were calculated using two-tailed unpaired Student’s *t*-test. shND1: shRNA expression vector targeting *NeuroD1*; pcCon: pcEF9-Puro; pcND1: *NeuroD1* overexpression vector; **P < 0.01.
(TIF)

S5 Fig *NeuroD1* overexpression abolished the effect of knocking down *NeuroD1* on ferroptosis. **(A)** Lipid peroxidation level in *NeuroD1* re-expressed *NeuroD1*-knocked down HCC-LM3 cells, as assessed using flow cytometry. Data were presented as % of oxidized/oxidized + reduced MFI (*F* value = 323.9). **(B)** Mitochondrial ROS level in *NeuroD1* re-expressed *NeuroD1*-knocked down HCC-LM3 cells, as assessed by Mito-SOX staining and flow cytometry (*F* value = 48.46). **(C–D)** MDA (C; *F* value = 21.57) and 4-HNE (D; *F* value = 37.89) levels in *NeuroD1* re-expressed *NeuroD1*-knocked down HCC-LM3 cells. **(E)** Level of LDH released from the *NeuroD1* re-expressed *NeuroD1*-knocked down HCC-LM3 cells (*F* value = 35.27). Cells transfected with shCon and/or pcCon were used as controls. Total protein was used for normalizing MDA and 4-HNE levels. Quantification data are expressed as mean ± SD (n = 3). One-way ANOVA and Tukey multiple comparisons analyses were performed when more than two groups were compared. shND1: shRNA expression vector targeting *NeuroD1*; pcCon: pcEF9-Puro; pcND1: *NeuroD1* overexpression vector; **P < 0.01.
(TIF)

S6 Fig NeuroD1 directly binds to the *GPX4* promoter and regulates its transcriptional activity in MHCC-97H cells. **(A–B)** Relative luciferase activities of GPX4-Luc-1 to GPX4-Luc-4 in *NeuroD1*-knocked down (A) and *NeuroD1*-overexpressed (B) MHCC-97H cells. **(C)** Binding capacity of NeuroD1 to the predicted region on the *GPX4* promoter in MHCC-97H cells, as examined using ChIP assay with anti-NeuroD1 antibody followed by PCR. Anti-histone H3 antibody was used as a positive control. Cells transfected with shCon or pcCon were used as controls. Quantification data are expressed as mean ± SD (n = 3). *P* values were calculated using two-tailed unpaired Student’s *t*-test. shND1: shRNA expression vector targeting *NeuroD1*; pcCon: pcEF9-Puro; pcND1: *NeuroD1* overexpression vector; **P < 0.01; NS: not significant.
(TIF)

S7 Fig NeuroD1/GPX4 axis regulates HCC cell viability and colony formation potential. **(A)** GPX4 protein expression levels in HCC-LM3 and MHCC-97H cells transfected with *GPX4* overexpression vector, as determined using western blotting. **(B)** NeuroD1 and GPX4 protein expression levels in *NeuroD1*-knocked down, *GPX4*-overexpressed HCC-LM3 cells, as determined using western blotting. **(C)** Viability of *NeuroD1*-knocked down, *GPX4*-overexpressed HCC-LM3 cells (*F* value = 292.2). **(D)** Colony formation potential of *NeuroD1*-knocked down, *GPX4*-overexpressed HCC-LM3 cells. Representative images (left) and quantification results (right) are shown (*F* value = 112.5). Cells transfected with shCon or pcCon were used as controls. β-actin was used as western blotting loading control. Quantification data are expressed as mean ± SD (n = 6). One-way ANOVA and Tukey multiple comparisons analyses were performed when more than two groups were compared. shND1: shRNA expression vector targeting *NeuroD1*; pcCon: pcEF9-Puro; **P < 0.01.
(TIF)

S8 Fig RSL-3 cancels the effect of NeuroD1 on promoting cell viability. **(A)** Lipid peroxidation level in *NeuroD1*-overexpressed HCC-LM3 cells treated with 5 μM RSL-3 for 24 h, as assessed using flow cytometry. Data were presented as % of oxidized/oxidized + reduced MFI (*F* value = 174.4). **(B)** Cell death rate in *NeuroD1*-overexpressed HCC-LM3 cells treated with 5 μM RSL-3 for 24 h, as assessed using PI staining and flow cytometry (*F* value = 734.3). **(C)** Viability of *NeuroD1*-overexpressed HCC-LM3 cells treated with indicated concentrations of RSL-3 for 36 h (n = 6). Cells transfected with pcCon or treated with DMSO were used as controls. Quantification data are expressed as mean ± SD (n = 3; unless otherwise indicated). *P* values were calculated using two-tailed unpaired Student’s *t*-test, or using one-way ANOVA and Tukey multiple comparisons when more than two groups were compared. pcCon: pcEF9-Puro; pcND1: *NeuroD1* overexpression vector; **P < 0.01.
(TIF)

S9 Fig mRNA expression levels of NeuroD1 and GPX4 in clinical tumor samples. **(A) NeuroD1 mRNA expression level in various tumors and the corresponding normal tissues (n = 256). (B) GPX4 mRNA level in various tumors and the corresponding normal tissues (n = 256).** *P* values were calculated using two-tailed unpaired Student’s *t*-test. Data was obtained from the TCGA and GTEx databases. **P < 0.01.
(TIF)

S10 Fig Establishment of *NeuroD1*-knocked down, *GPX4*-overexpressed HCC-LM3 stable cell line. **NeuroD1 and GPX4 protein expression levels in *NeuroD1*-knocked down, *GPX4*-overexpressed HCC-LM3 stable cell line, as determined using western blotting.** β-actin was used as western blotting loading control.
(TIF)

S1 Table Primer pairs used for qRT-PCR. (PDF)

S2 Table Antibodies used for western blotting, immunofluorescence, ChIP assay, immunoprecipitation, and immunohistochemistry. (PDF)

S1 Data All numerical and statistical data for quantitation figures. (XLSX)
