# Supplementary figures and images for: NeuroD1-GPX4 signaling leads to ferroptosis resistance in hepatocellular carcinoma
Source: PLoS Genet. 2023 Dec 22;19(12):e1011098. doi: 10.1371/journal.pgen.1011098 (PMC10773945; doi:10.1371/journal.pgen.1011098)

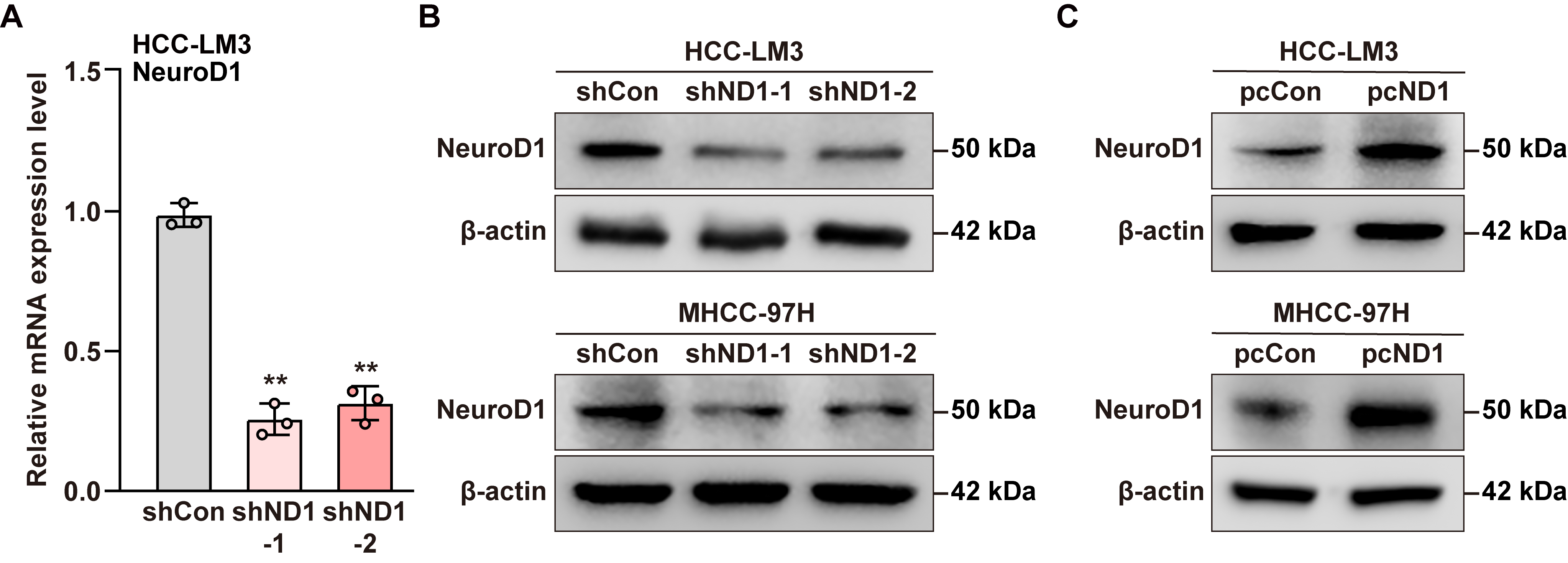

Supplement: S1 Fig — (A) NeuroD1 mRNA expression level in HCC-LM3 cells transfected with two shRNA expression vectors targeting different sites of NeuroD1, as determined using qRT-PCR. (B–C) NeuroD1 protein expression levels in HCC-LM3 and MHCC-97H cells transfected with two shRNA expression vectors targeting different sites of NeuroD1 (B) and NeuroD1 overexpression vector (C), as determined using western blotting. Cells transfected with shCon or pcCon were used as controls. β-actin was used for qRT-PCR normalization and as western blotting loading control. Quantification data are expressed as mean ± SD (n = 3). P values were calculated using two-tailed unpaired Student’s t-test. shND1: shRNA expression vector targeting NeuroD1; pcCon: pcEF9-Puro; pcND1: NeuroD1 overexpression vector; **P < 0.01. (TIF) [file pgen.1011098.s001.tif]

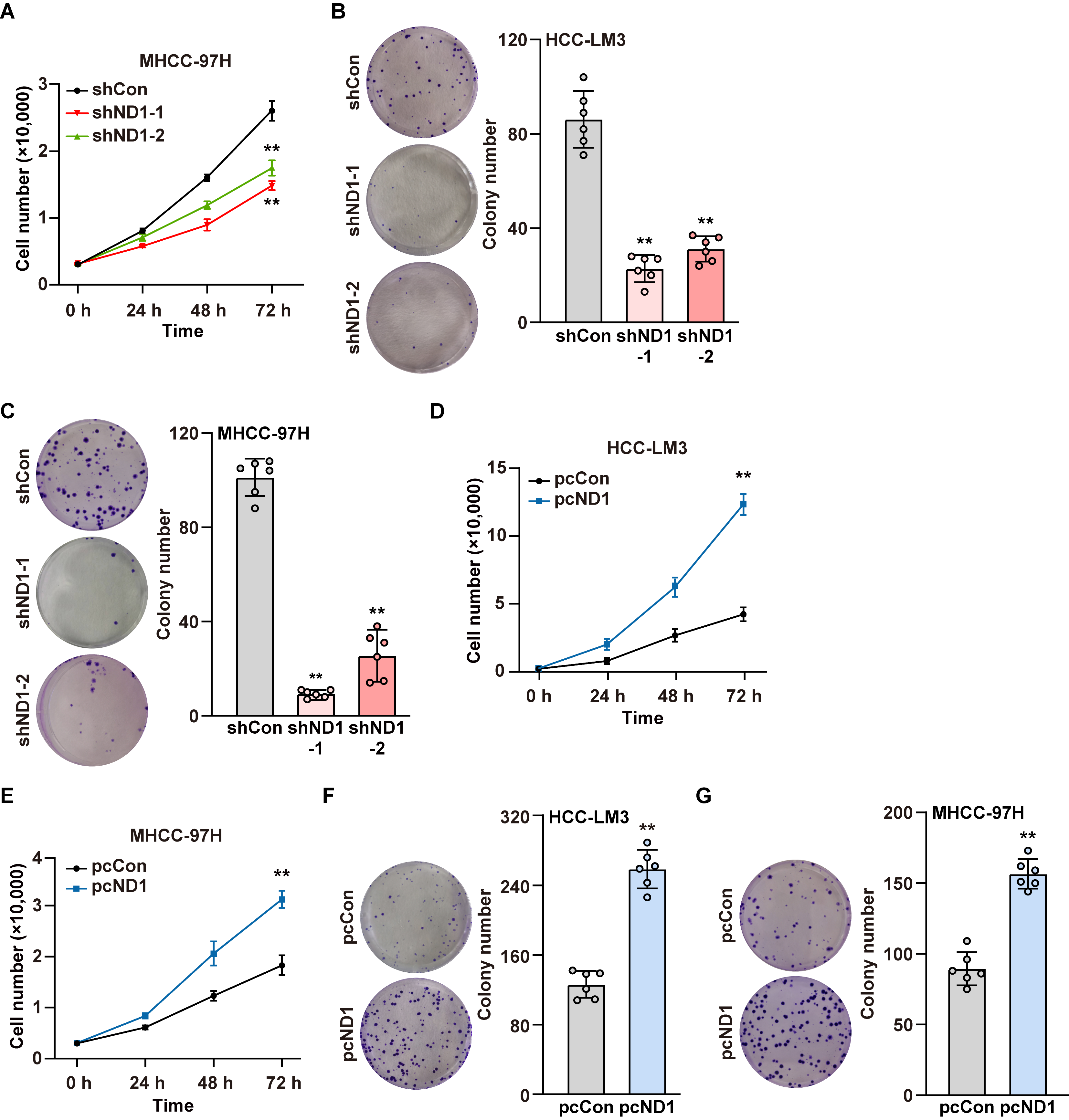

Supplement: S2 Fig — (A) Viability of NeuroD1-knocked down MHCC-97Hcells. (B–C) Colony formation potential of NeuroD1-knocked down HCC-LM3 (B) and MHCC-97H (C) cells. Representative images (left) and quantification results (right) are shown. (D–E) Viability of NeuroD1-overexpressed HCC-LM3 (D) and MHCC-97H (E) cells. (F–G) Colony formation potential of NeuroD1-overexpressed HCC-LM3 (F) and MHCC-97H (G) cells. Representative images (left) and quantification results (right) are shown. Cells transfected with shCon or pcCon were used as controls. Quantification data are expressed as mean ± SD (n = 6). P values were calculated using two-tailed unpaired Student’s t-test. shND1: shRNA expression vector targeting NeuroD1; pcCon: pcEF9-Puro; pcND1: NeuroD1 overexpression vector; **P < 0.01. (TIF) [file pgen.1011098.s002.tif]

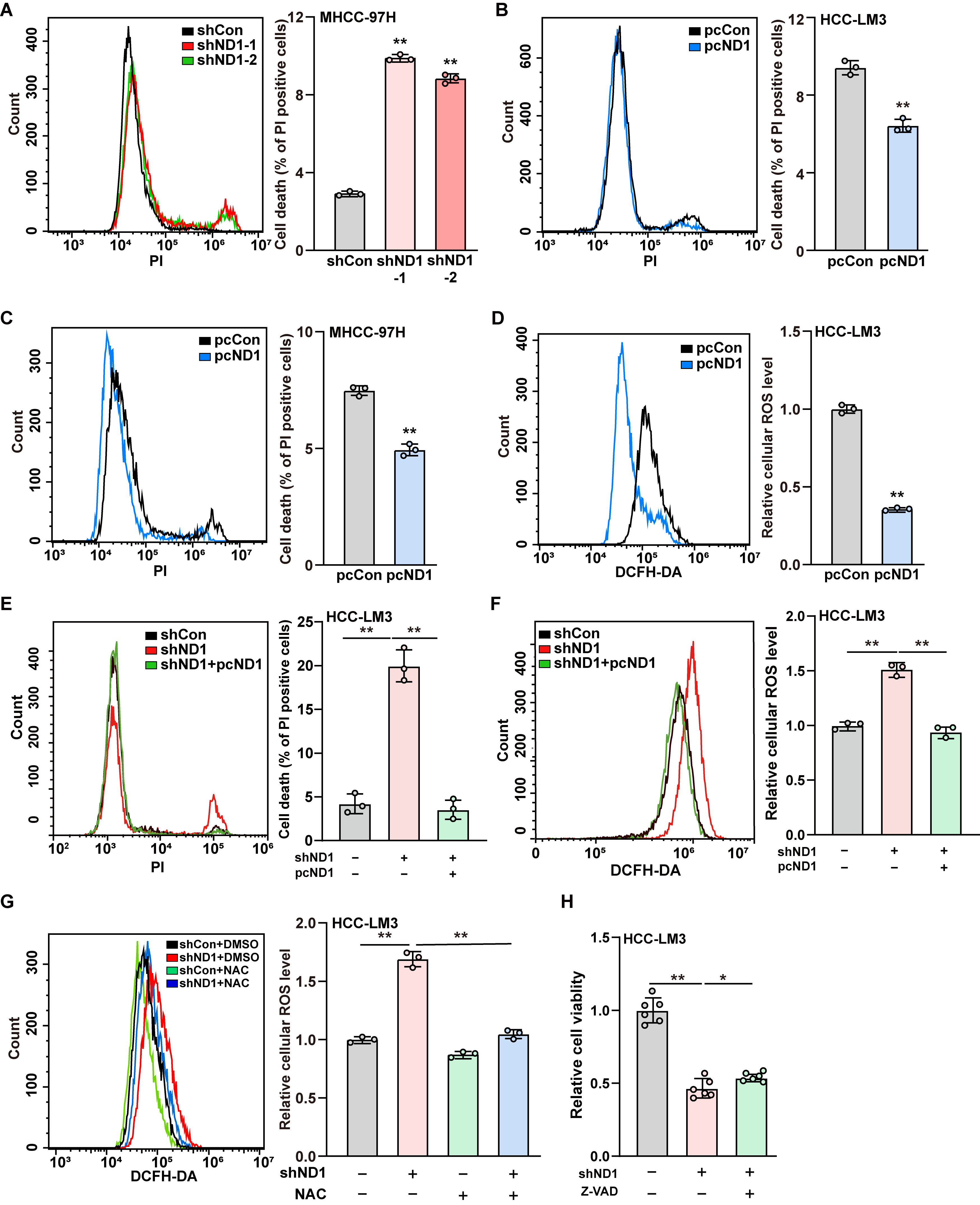

Supplement: S3 Fig — (A) Cell death rate of NeuroD1-knocked down MHCC-97H cells, as examined using PI staining and flow cytometry. (B–C) Cell death rate of NeuroD1-overexpressed HCC-LM3 cells (B) and MHCC-97H cells (C), as examined using PI staining and flow cytometry. (D) Total cellular ROS level in NeuroD1-overexpressed HCC-LM3 cells. (E) Cell death rate of NeuroD1 re-expressed NeuroD1-knocked down HCC-LM3 cells, as examined using PI staining and flow cytometry (F value = 134.8). (F) Total cellular ROS level in NeuroD1 re-expressed NeuroD1-knocked down HCC-LM3 cells, as assessed using DCFH-DA staining and flow cytometry (F value = 133.9). (G) Total cellular ROS level in NeuroD1 knocked down, N-acetylcysteine (NAC)-treated HCC-LM3 cells, as assessed using DCFH-DA staining and flow cytometry (F value = 234.1). (H) Relative viability of NeuroD1-knocked down HCC-LM3 cells treated with Z-VAD (final concentration: 20 μM) for 48 h (n = 6; F value = 109.3). Cells transfected with shCon, pcCon, or treated with DMSO were used as controls. Quantification data are expressed as mean ± SD (n = 3). P values were calculated using two-tailed unpaired Student’s t-test, or using one-way ANOVA and Tukey multiple comparisons when more than two groups were compared. shND1: shRNA expression vector targeting NeuroD1; pcCon: pcEF9-Puro; pcND1: NeuroD1 overexpression vector; *P < 0.05, **P < 0.01. (TIF) [file pgen.1011098.s003.tif]

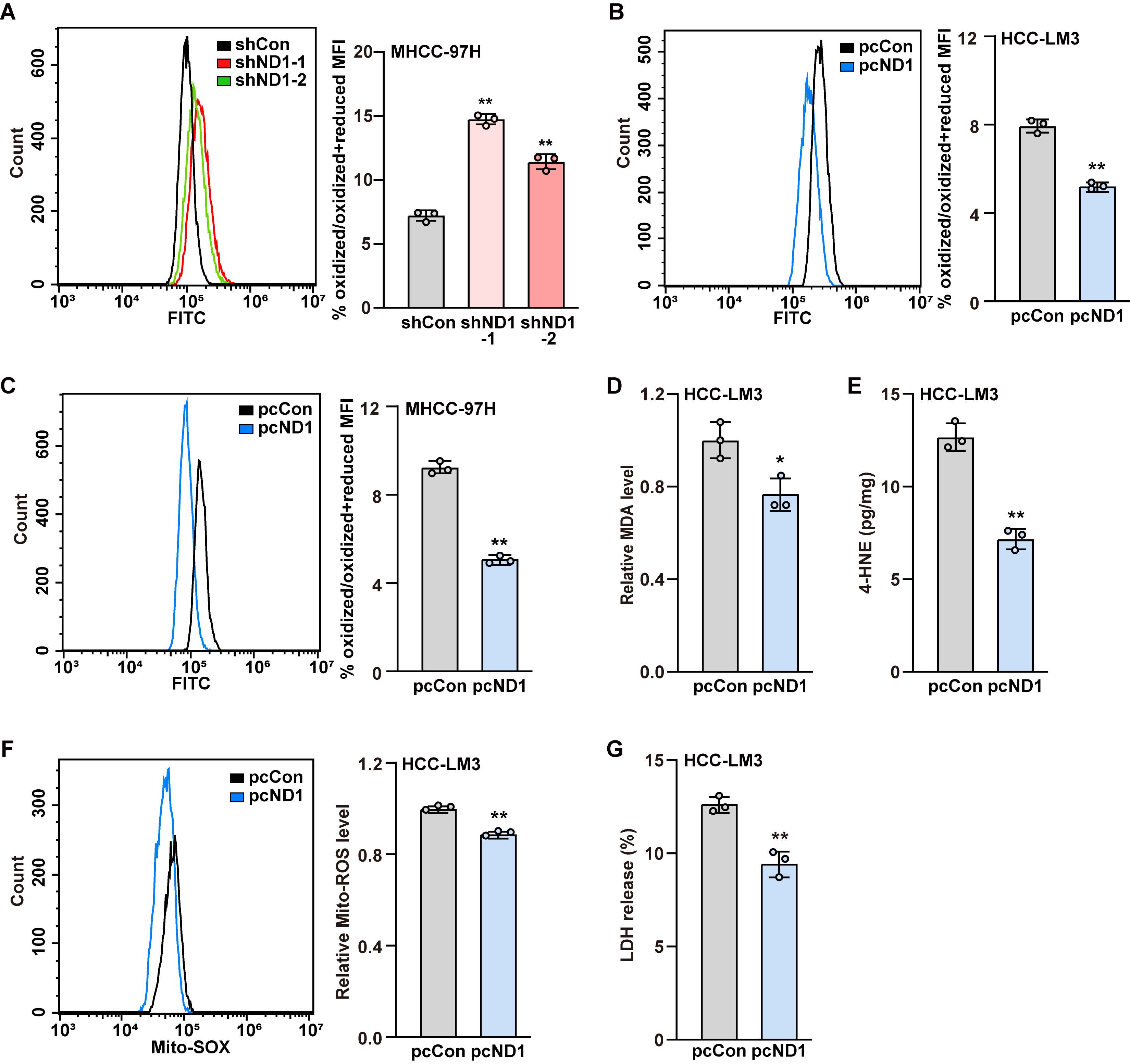

Supplement: S4 Fig — (A) Lipid peroxidation level in NeuroD1-knocked down MHCC-97H cells, as assessed using flow cytometry. Data were presented as % of oxidized/oxidized + reduced MFI. (B–C) Lipid peroxidation level in NeuroD1-overexpressed HCC-LM3 (B) and MHCC-97H (C) cells, as assessed using flow cytometry. Data were presented as % of oxidized/oxidized + reduced MFI. (D–E) MDA (D) and 4-HNE (E) levels in NeuroD1-overexpressed HCC-LM3 cells. (F) Mitochondrial ROS level in NeuroD1-overexpressed HCC-LM3 cells, as assessed by Mito-SOX staining and flow cytometry. (G) Level of LDH released from NeuroD1-overexpressed HCC-LM3 cells. Cells transfected with shCon or pcCon were used as controls. Total protein was used for normalizing MDA and 4-HNE levels. Quantification data are expressed as mean ± SD (n = 3). P values were calculated using two-tailed unpaired Student’s t-test. shND1: shRNA expression vector targeting NeuroD1; pcCon: pcEF9-Puro; pcND1: NeuroD1 overexpression vector; **P < 0.01. (TIF) [file pgen.1011098.s004.tif]

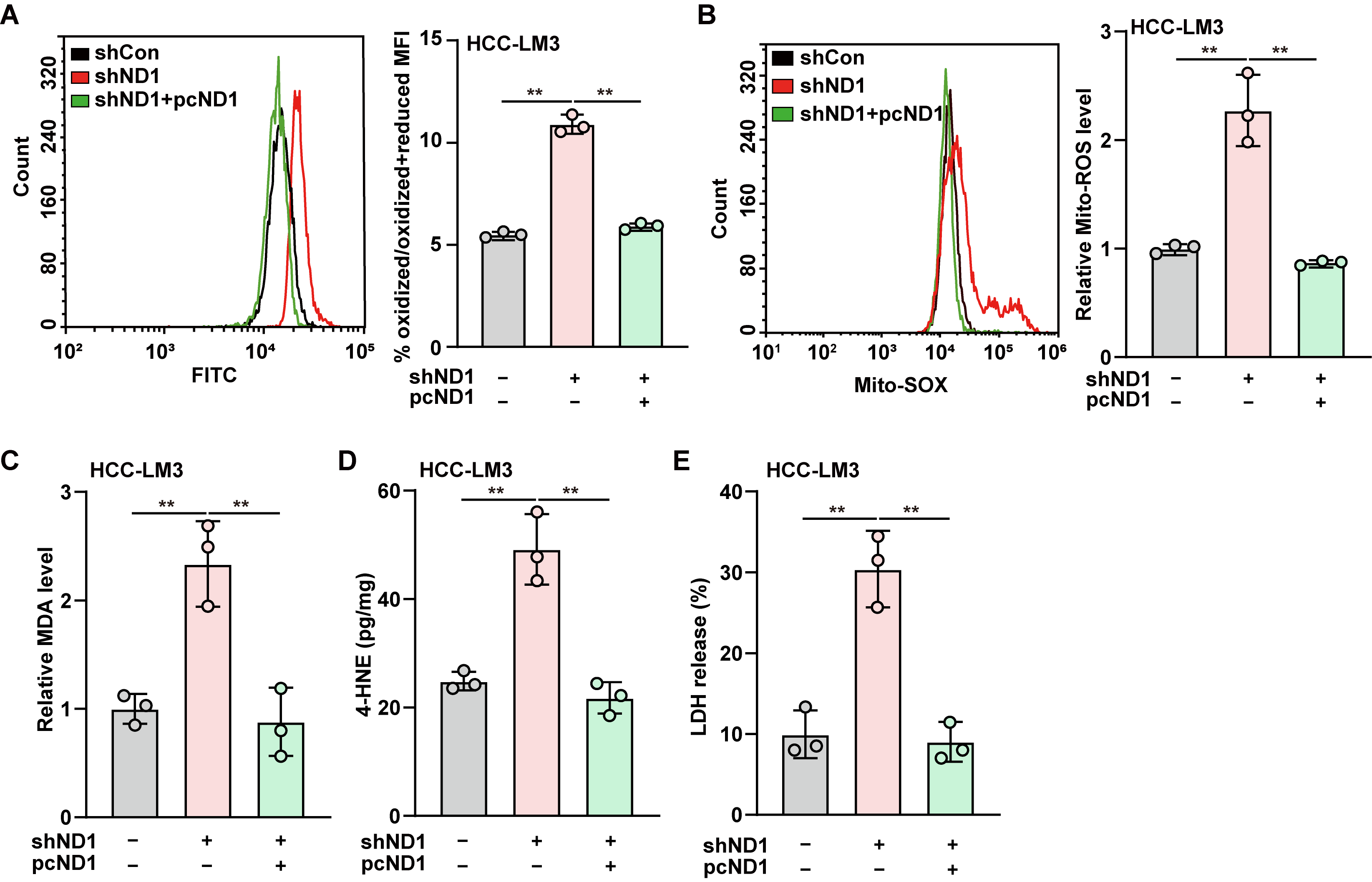

Supplement: S5 Fig — (A) Lipid peroxidation level in NeuroD1 re-expressed NeuroD1-knocked down HCC-LM3 cells, as assessed using flow cytometry. Data were presented as % of oxidized/oxidized + reduced MFI (F value = 323.9). (B) Mitochondrial ROS level in NeuroD1 re-expressed NeuroD1-knocked down HCC-LM3 cells, as assessed by Mito-SOX staining and flow cytometry (F value = 48.46). (C–D) MDA (C; F value = 21.57) and 4-HNE (D; F value = 37.89) levels in NeuroD1 re-expressed NeuroD1-knocked down HCC-LM3 cells. (E) Level of LDH released from the NeuroD1 re-expressed NeuroD1-knocked down HCC-LM3 cells (F value = 35.27). Cells transfected with shCon and/or pcCon were used as controls. Total protein was used for normalizing MDA and 4-HNE levels. Quantification data are expressed as mean ± SD (n = 3). One-way ANOVA and Tukey multiple comparisons analyses were performed when more than two groups were compared. shND1: shRNA expression vector targeting NeuroD1; pcCon: pcEF9-Puro; pcND1: NeuroD1 overexpression vector; **P < 0.01. (TIF) [file pgen.1011098.s005.tif]

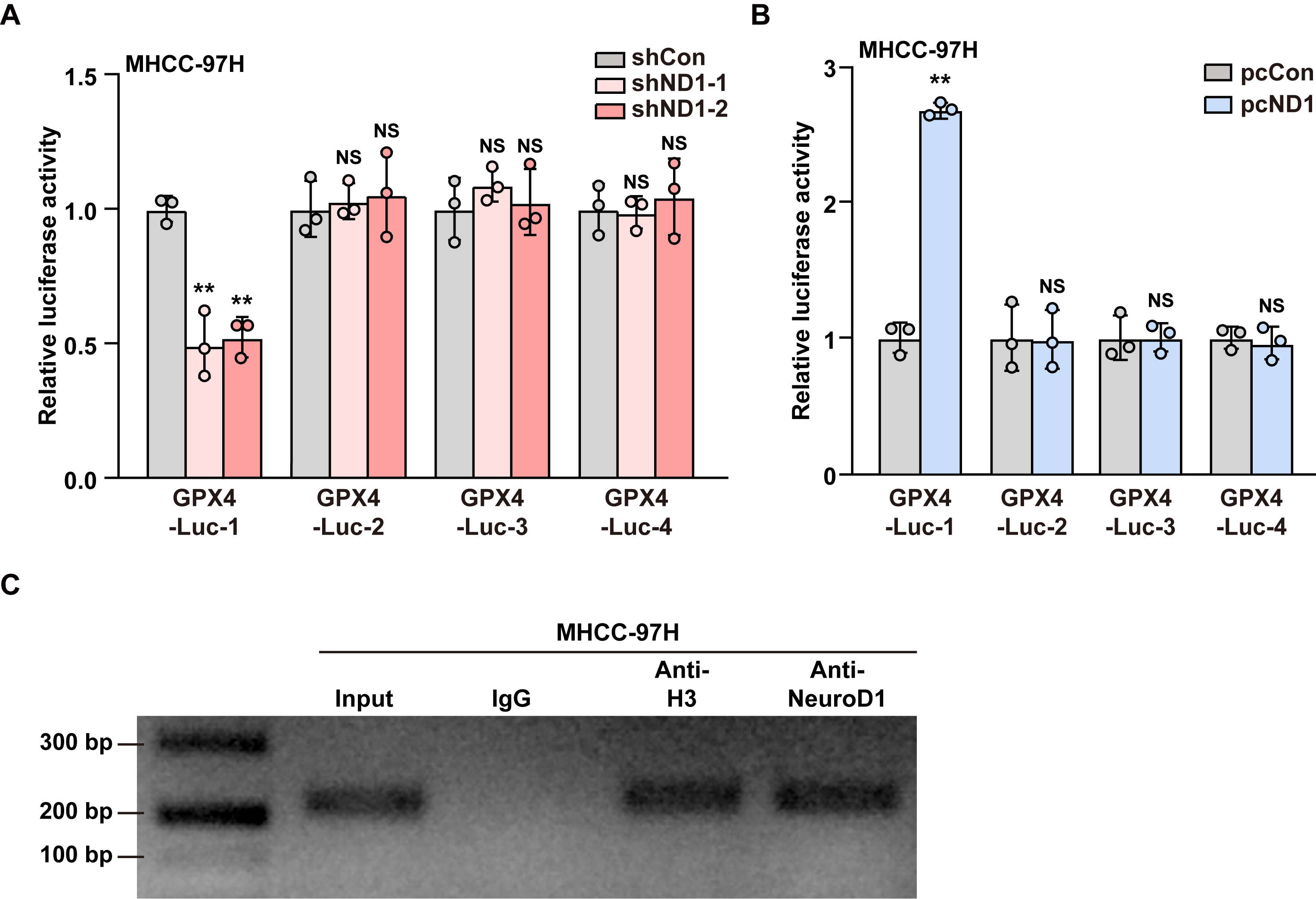

Supplement: S6 Fig — (A–B) Relative luciferase activities of GPX4-Luc-1 to GPX4-Luc-4 in NeuroD1-knocked down (A) and NeuroD1-overexpressed (B) MHCC-97H cells. (C) Binding capacity of NeuroD1 to the predicted region on the GPX4 promoter in MHCC-97H cells, as examined using ChIP assay with anti-NeuroD1 antibody followed by PCR. Anti-histone H3 antibody was used as a positive control. Cells transfected with shCon or pcCon were used as controls. Quantification data are expressed as mean ± SD (n = 3). P values were calculated using two-tailed unpaired Student’s t-test. shND1: shRNA expression vector targeting NeuroD1; pcCon: pcEF9-Puro; pcND1: NeuroD1 overexpression vector; **P < 0.01; NS: not significant. (TIF) [file pgen.1011098.s006.tif]

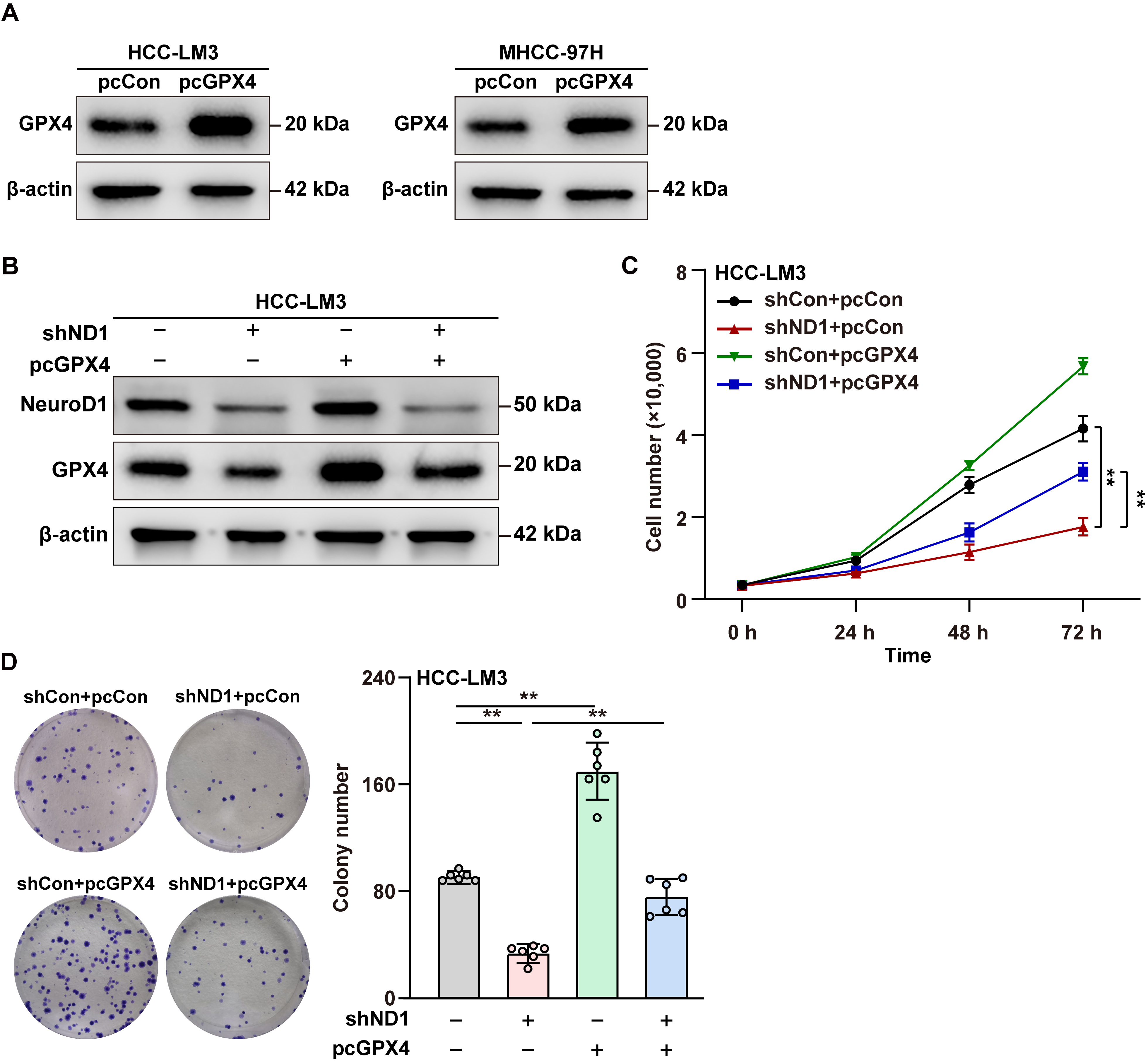

Supplement: S7 Fig — (A) GPX4 protein expression levels in HCC-LM3 and MHCC-97H cells transfected with GPX4 overexpression vector, as determined using western blotting. (B) NeuroD1 and GPX4 protein expression levels in NeuroD1-knocked down, GPX4-overexpressed HCC-LM3 cells, as determined using western blotting. (C) Viability of NeuroD1-knocked down, GPX4-overexpressed HCC-LM3 cells (F value = 292.2). (D) Colony formation potential of NeuroD1-knocked down, GPX4-overexpressed HCC-LM3 cells. Representative images (left) and quantification results (right) are shown (F value = 112.5). Cells transfected with shCon or pcCon were used as controls. β-actin was used as western blotting loading control. Quantification data are expressed as mean ± SD (n = 6). One-way ANOVA and Tukey multiple comparisons analyses were performed when more than two groups were compared. shND1: shRNA expression vector targeting NeuroD1; pcCon: pcEF9-Puro; **P < 0.01. (TIF) [file pgen.1011098.s007.tif]

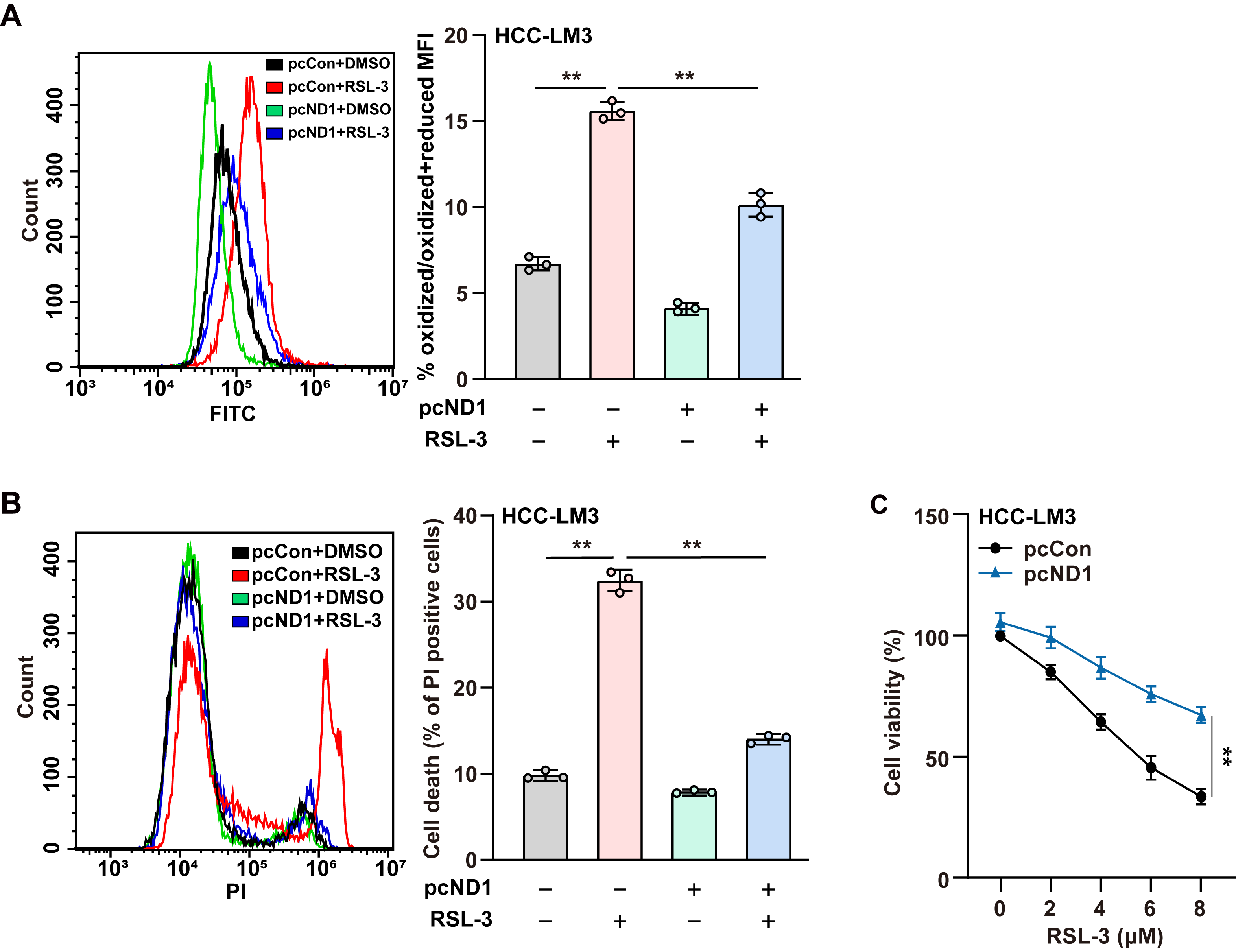

Supplement: S8 Fig — (A) Lipid peroxidation level in NeuroD1-overexpressed HCC-LM3 cells treated with 5 μM RSL-3 for 24 h, as assessed using flow cytometry. Data were presented as % of oxidized/oxidized + reduced MFI (F value = 174.4). (B) Cell death rate in NeuroD1-overexpressed HCC-LM3 cells treated with 5 μM RSL-3 for 24 h, as assessed using PI staining and flow cytometry (F value = 734.3). (C) Viability of NeuroD1-overexpressed HCC-LM3 cells treated with indicated concentrations of RSL-3 for 36 h (n = 6). Cells transfected with pcCon or treated with DMSO were used as controls. Quantification data are expressed as mean ± SD (n = 3; unless otherwise indicated). P values were calculated using two-tailed unpaired Student’s t-test, or using one-way ANOVA and Tukey multiple comparisons when more than two groups were compared. pcCon: pcEF9-Puro; pcND1: NeuroD1 overexpression vector; **P < 0.01. (TIF) [file pgen.1011098.s008.tif]

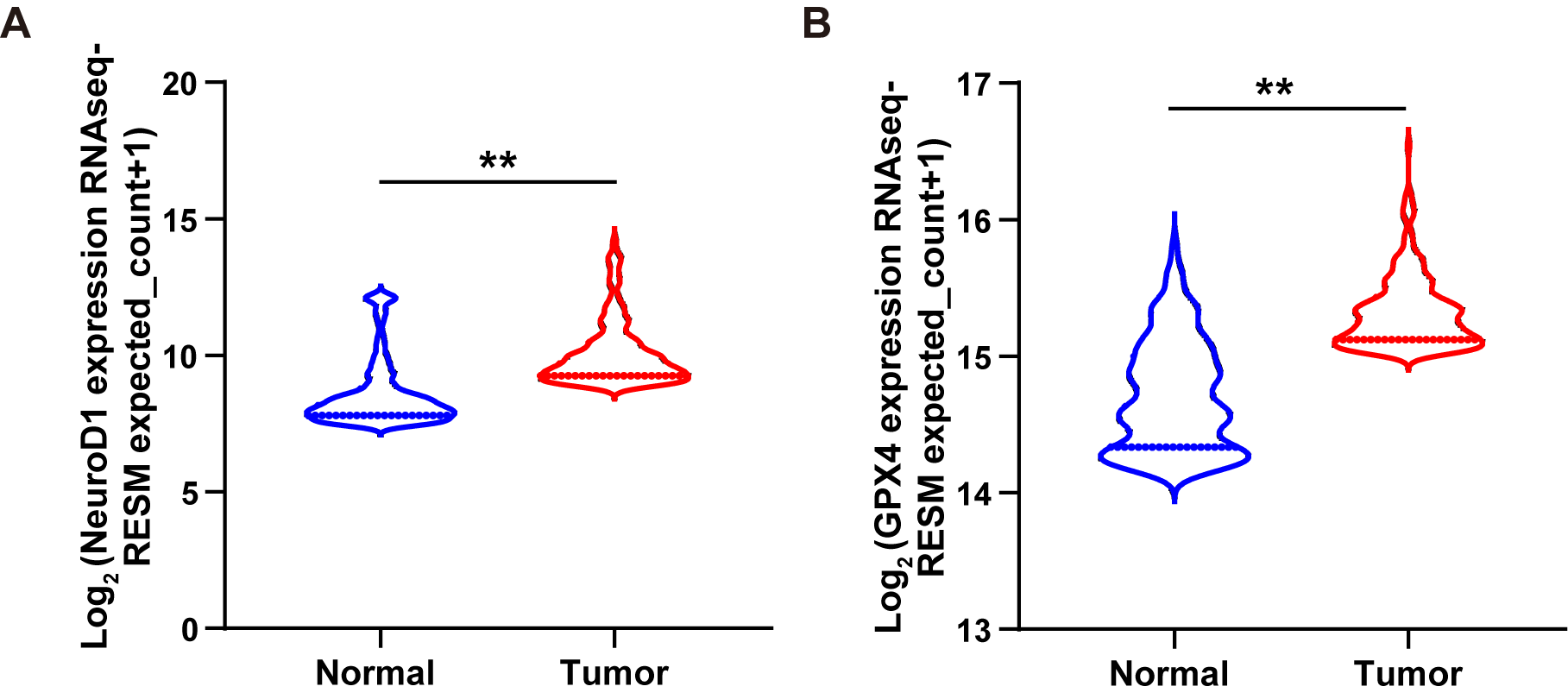

Supplement: S9 Fig — (A) NeuroD1 mRNA expression level in various tumors and the corresponding normal tissues (n = 256). (B) GPX4 mRNA level in various tumors and the corresponding normal tissues (n = 256). P values were calculated using two-tailed unpaired Student’s t-test. Data was obtained from the TCGA and GTEx databases. **P < 0.01. (TIF) [file pgen.1011098.s009.tif]

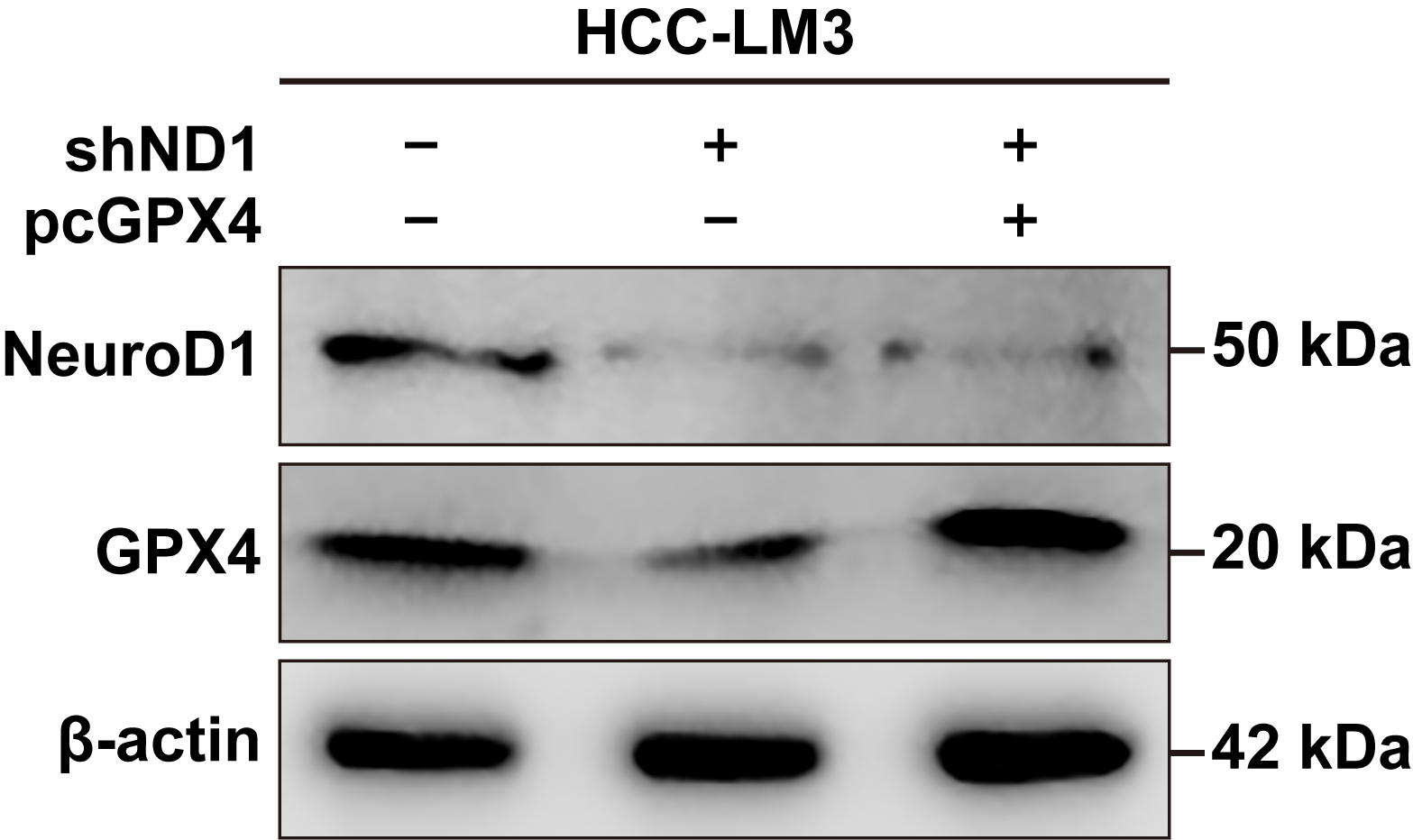

Supplement: S10 Fig — NeuroD1 and GPX4 protein expression levels in NeuroD1-knocked down, GPX4-overexpressed HCC-LM3 stable cell line, as determined using western blotting. β-actin was used as western blotting loading control. (TIF) [file pgen.1011098.s010.tif]
