# Supplementary material for: NeuroD1-GPX4 signaling leads to ferroptosis resistance in hepatocellular carcinoma
Source: PLoS Genet. 2023 Dec 22;19(12):e1011098. doi: 10.1371/journal.pgen.1011098 (PMC10773945; doi:10.1371/journal.pgen.1011098)
Supplement: S1 Table — (PDF) [file pgen.1011098.s011.pdf]

S1 Table. Primer pairs used for qRT-PCR.

Genes	Refseq No.	Primer sequences (5'-3')	
		Forward	Reverse
β-actin	NM_001101.5	TCCCTGGAGAAGAGCTACG	GTAGTTTCGTGGATGCCACA
NeuroD1	NM_002500.5	ACCTGGTCTCCTTCGTTCAG	ACTGGTAGGAGTAGGGGTGT
GPX4	NM_001367832.1	CGCTGTGGAAGTGGATGAA	TTGTGGAGCTAGAAATAGTGGG
ALOX12	NM_000697.3	CCAAAGGGATGACATAGTGAA	GGTGAGGAAATGGCAGAGTT
FDFT1	NM_001287742.2	GAGGTTTGGAGCAGGTAT	TGAGTCTCGAAAGGTAGGT
LPCAT3	NM_005768.6	TCTGGCTGGATACTATTACAC	CACGTATGGCATATTTCTG
NCOA4	NM_001145260.2	GCTTGCTATTGGTGGAGT	AGCCTGCTGTTGAAGTGTCT
NFE2L2	NM_001145412.3	AGCGACGGAAAGAGTATGA	TGGGAGTAGTTGGCAGAT
