# Supplementary material for: NeuroD1-GPX4 signaling leads to ferroptosis resistance in hepatocellular carcinoma
Source: PLoS Genet. 2023 Dec 22;19(12):e1011098. doi: 10.1371/journal.pgen.1011098 (PMC10773945; doi:10.1371/journal.pgen.1011098)
Supplement: S2 Table — (PDF) [file pgen.1011098.s012.pdf]

S2 Table. Antibodies used for western blotting, immunofluorescence, ChIP assay, immunoprecipitation, and immunohistochemistry.

Antibody	Maker	Product No.	Experiment	Dilution
Anti-β-actin	Proteintech	60008-1- Ig	Western blotting	1/10,000
Anti-NeuroD1	Proteintech	12081-1-AP	Western blotting<br>ChIP assay<br>Immunoprecipitation<br>Immunohistochemistry	1/1,000<br>30 μg/mL cell lysate<br>30 μg/mL cell lysate<br>1/200
Anti-GPX4	Proteintech	67763-1-Ig	Western blotting<br>Immunohistochemistry	1/4,000<br>1/1,000
Anti-RNA Polymerase II	ZEN BIO	R26223	Western blotting	1/1,000
Anti-4HNE	Bioss	Bs-6313R	Immunohistochemistry	1/200
Anti-Histone H3	Proteintech	17168-1-AP	ChIP assay	30 μg/mL cell lysate
Anti-Rabbit IgG	Proteintech	B900610	ChIP assay	30 μg/mL cell lysate
Goat Anti-Rabbit IgG	ZSGB-BIO	ZB2301	Western blotting	1/10,000
Goat Anti-Mouse IgG	ZSGB-BIO	ZB2305	Western blotting	1/10,000
Anti-γ-H2AX	Abcam	ab81299	Immunofluorescence	1/250
Alexa Fluor 488 Goat Anti-Rat IgG	Invitrogen	A-21206	Immunofluorescence	1/800
